# Supplementary material for: Caspar specifies primordial germ cell count and identity in Drosophila melanogaster
Source: eLife. 2024 Dec 13;13:RP98584. doi: 10.7554/eLife.98584 (PMC11643641; doi:10.7554/eLife.98584)
Supplement: Figure 1—source data 1. [file elife-98584-fig1-data1.pdf]

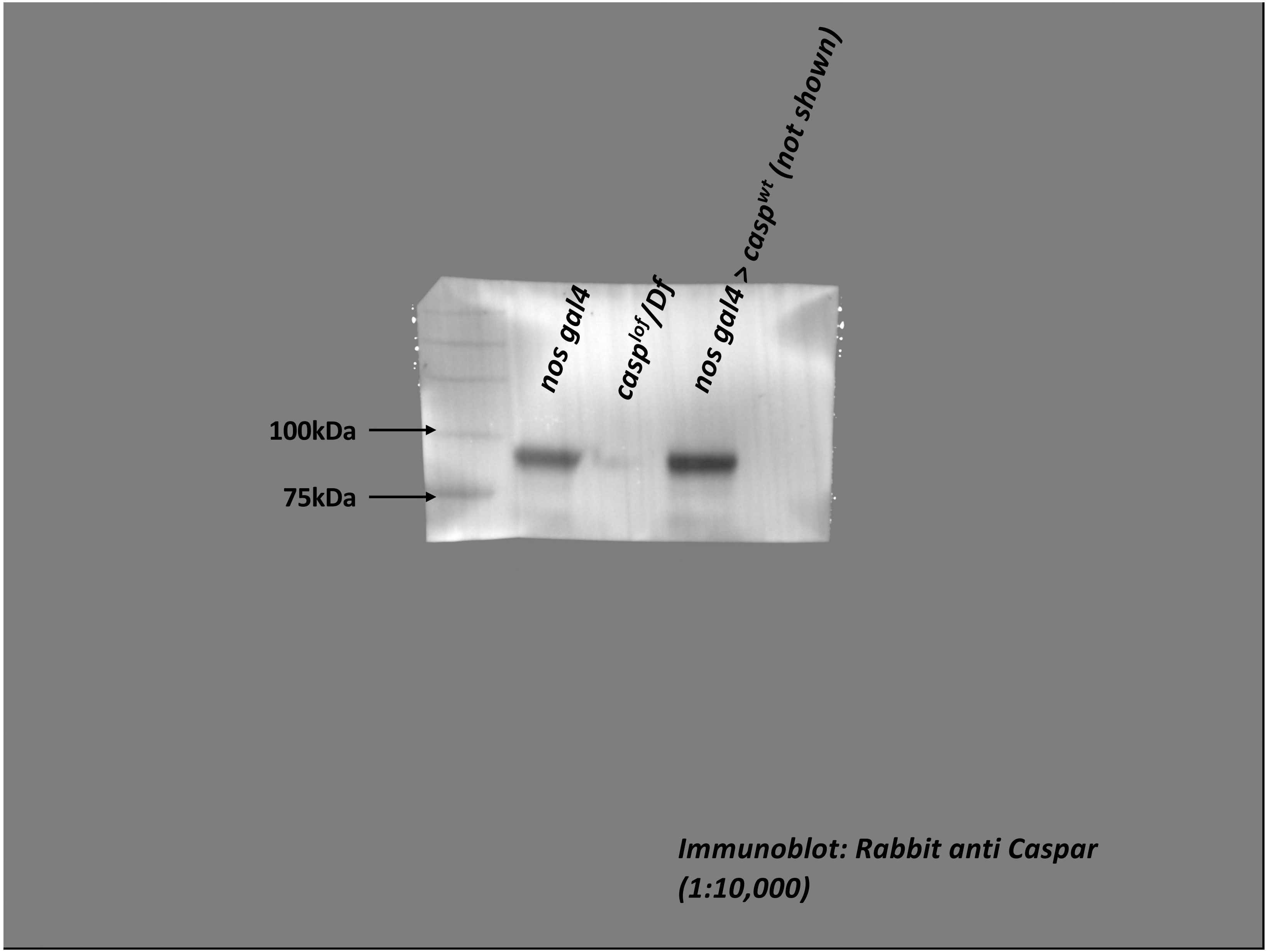

Figure 1, Source Data 1. Original membranes corresponding to Figure 1, panel E. Blot was probed with rabbit anti-Caspar antibody. Biorad Precision Plus Protein standards (Dual Colour) was used as molecular weight marker

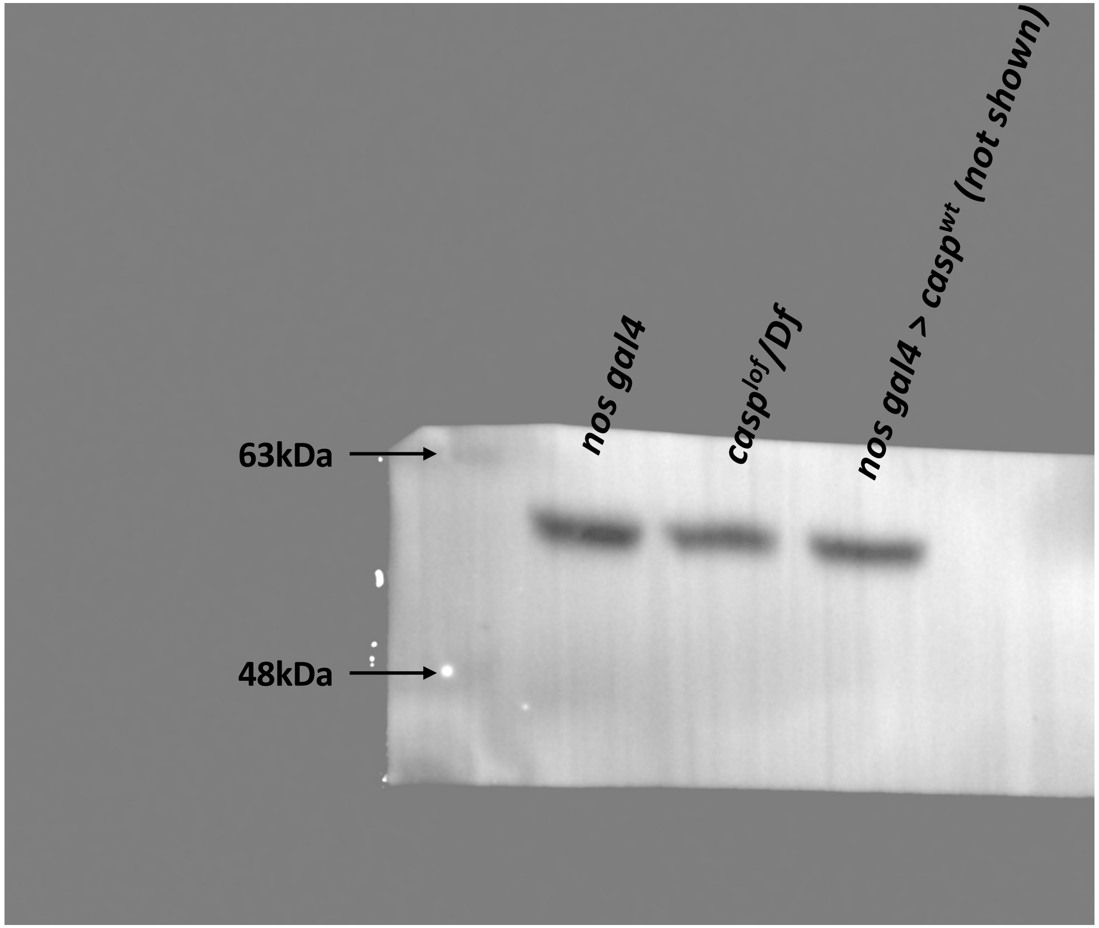

Immunoblot: Mouse anti Tubulin (1:10,000)

Figure 1, Source Data 1. Original membranes corresponding to Figure 1, panel E. Blot was probed with mouse anti-Tubulin antibody. Biorad Precision Plus Protein standards (Dual Colour) was used as molecular weight marker
