# Supplementary figures and images for: Caspar specifies primordial germ cell count and identity in Drosophila melanogaster
Source: eLife. 2024 Dec 13;13:RP98584. doi: 10.7554/eLife.98584 (PMC11643641; doi:10.7554/eLife.98584)

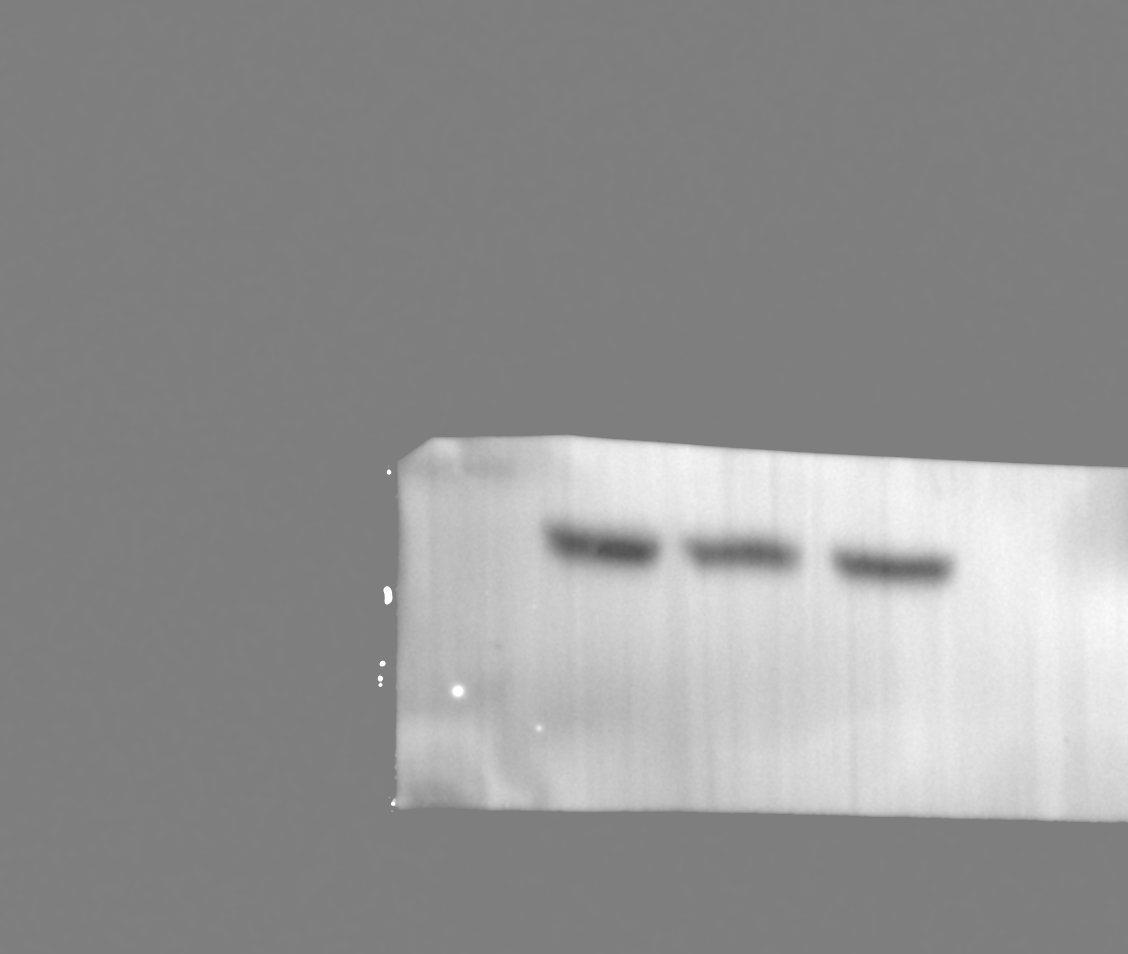

Supplement: Figure 1—source data 2. [file elife-98584-fig1-data2.zip › Figure 1 source data 2/Tubulin.tif]

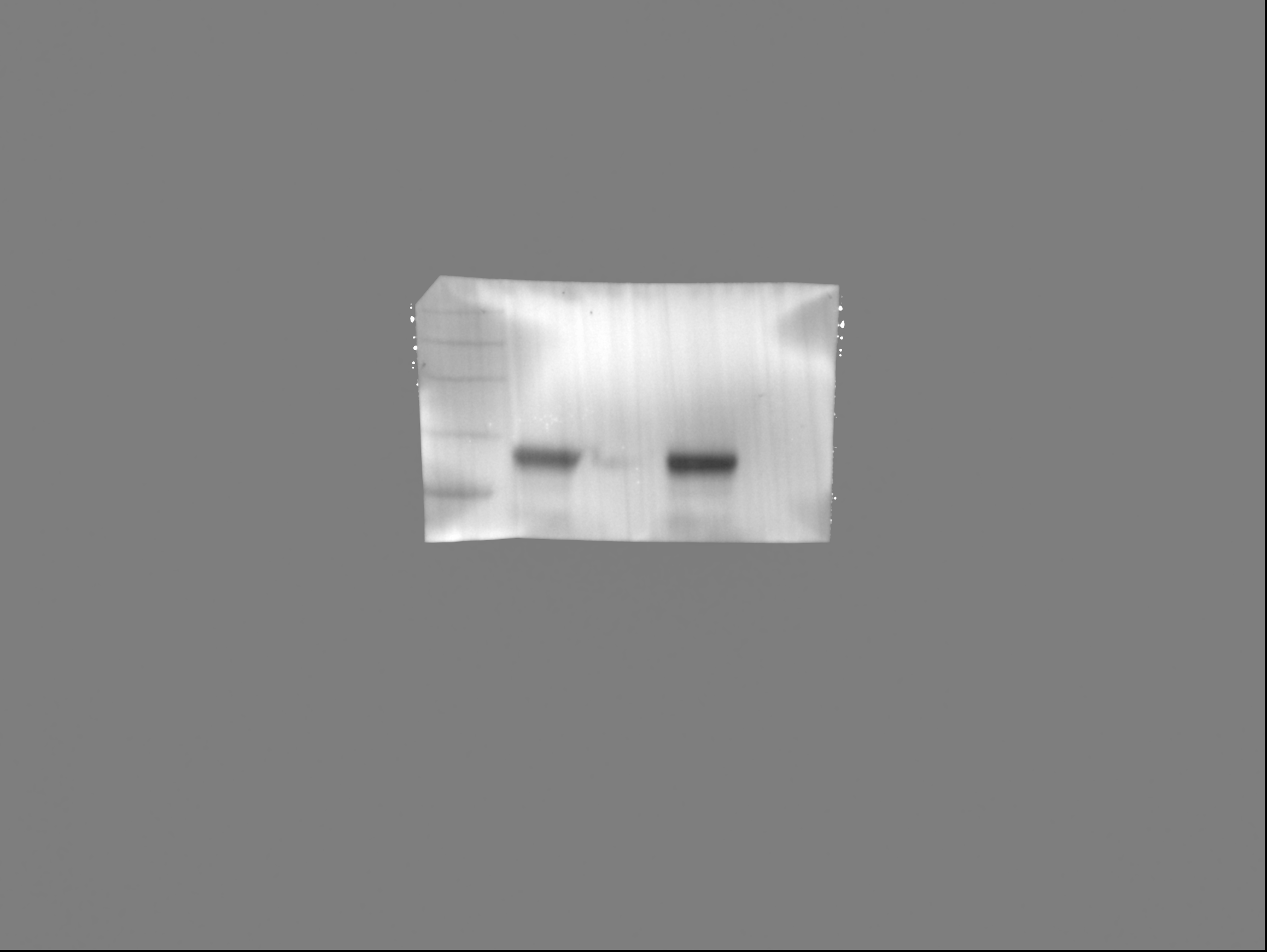

Supplement: Figure 1—source data 2. [file elife-98584-fig1-data2.zip › Figure 1 source data 2/Caspar.tif]

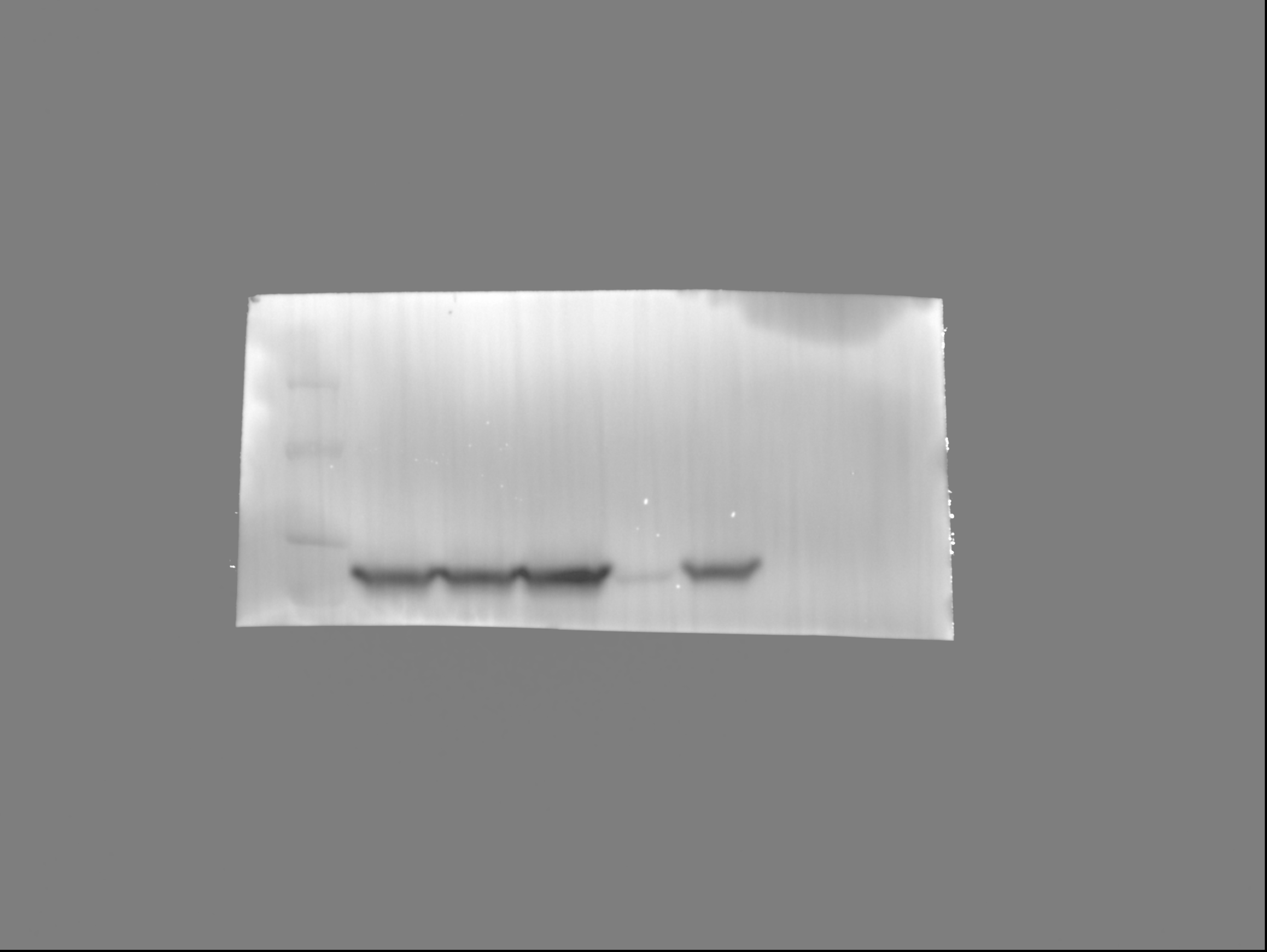

Supplement: Figure 3—source data 2. [file elife-98584-fig3-data2.zip › Figure 3- Source data 1/Caspar.tif]

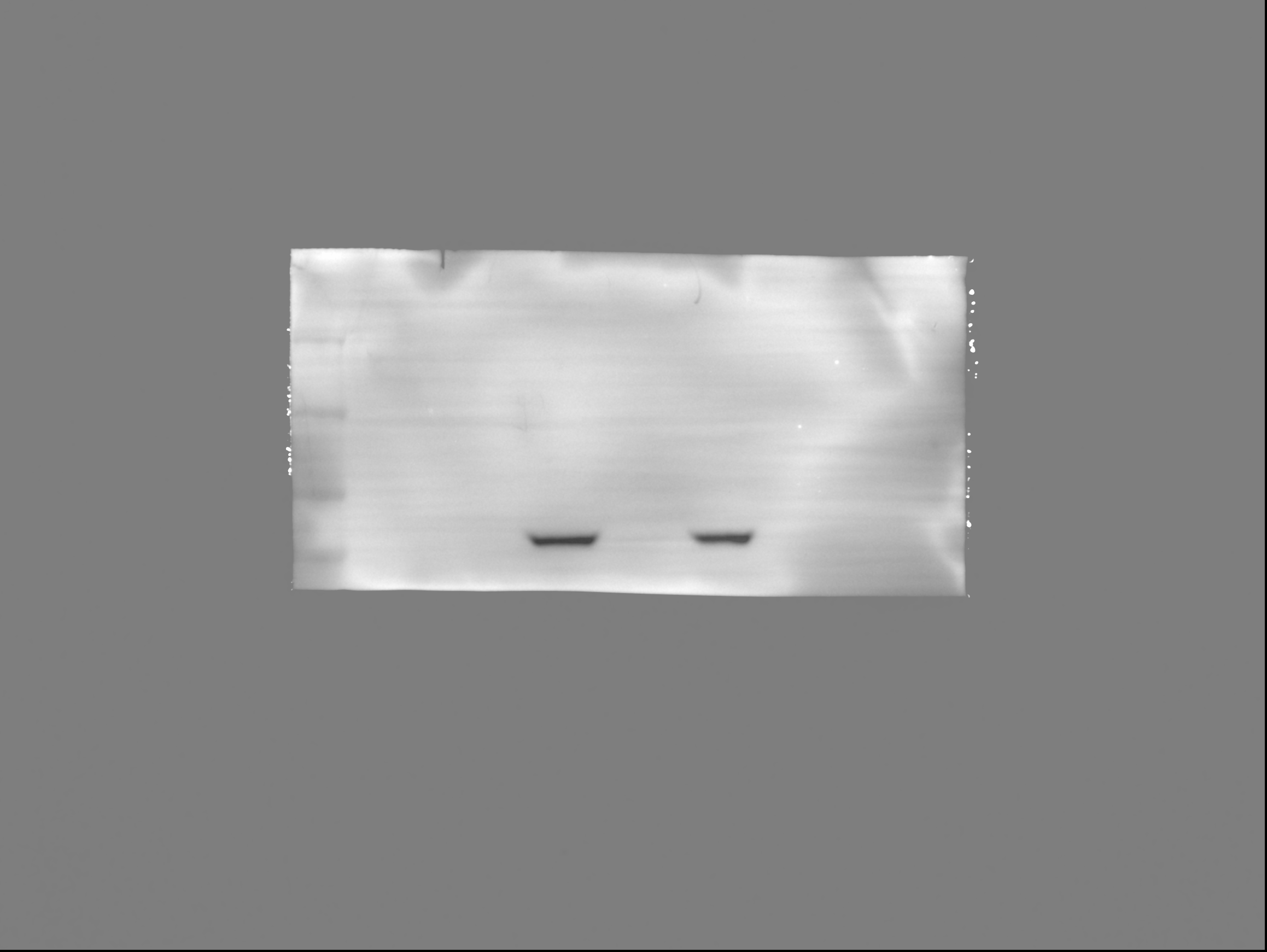

Supplement: Figure 3—source data 2. [file elife-98584-fig3-data2.zip › Figure 3- Source data 1/HA.tif]

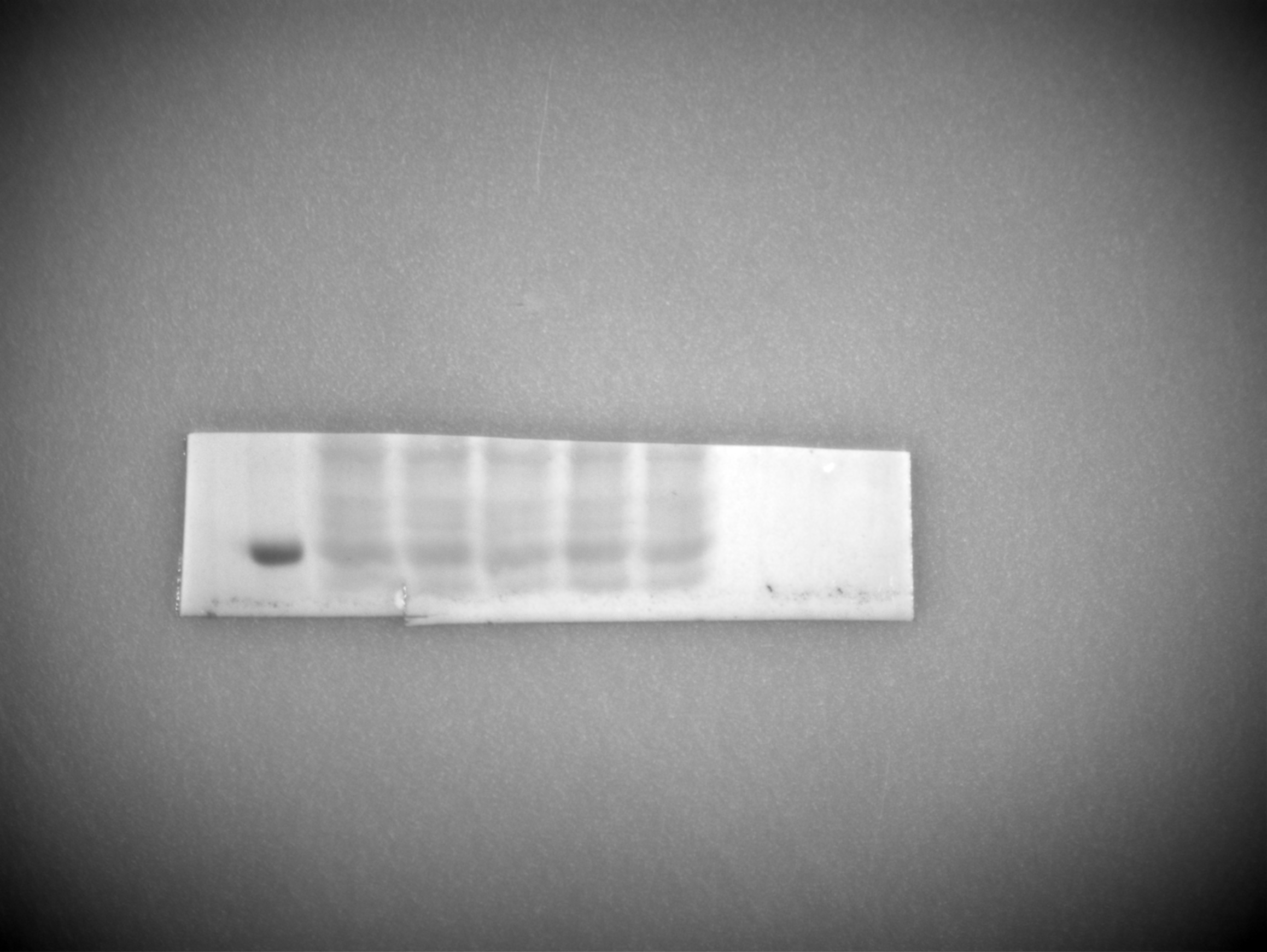

Supplement: Figure 3—source data 2. [file elife-98584-fig3-data2.zip › Figure 3- Source data 1/ponceau.tif]

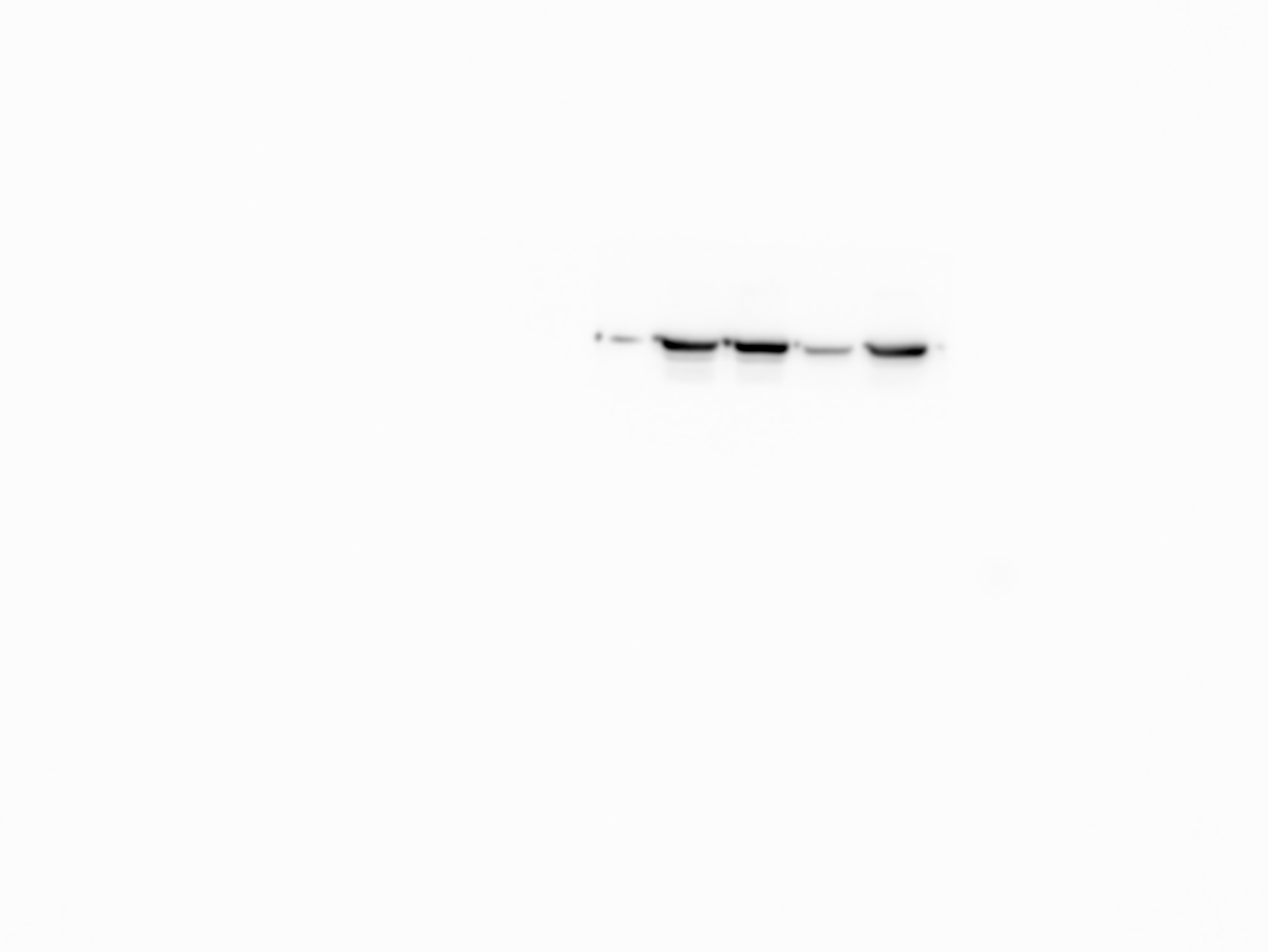

Supplement: Figure 4—source data 2. [file elife-98584-fig4-data2.zip › Figure 4 - Source Data 1/1_VCP WB w lof teri culli 16092021.tif]

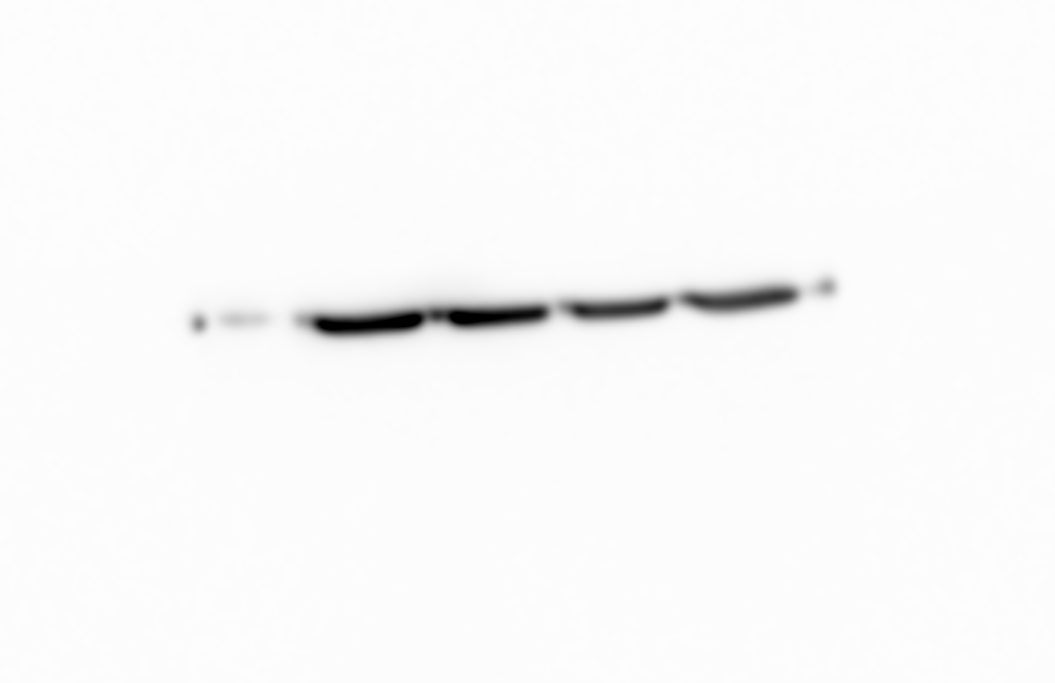

Supplement: Figure 4—source data 2. [file elife-98584-fig4-data2.zip › Figure 4 - Source Data 1/3_tub for BiP VCP w lof teri 16092021.tif]

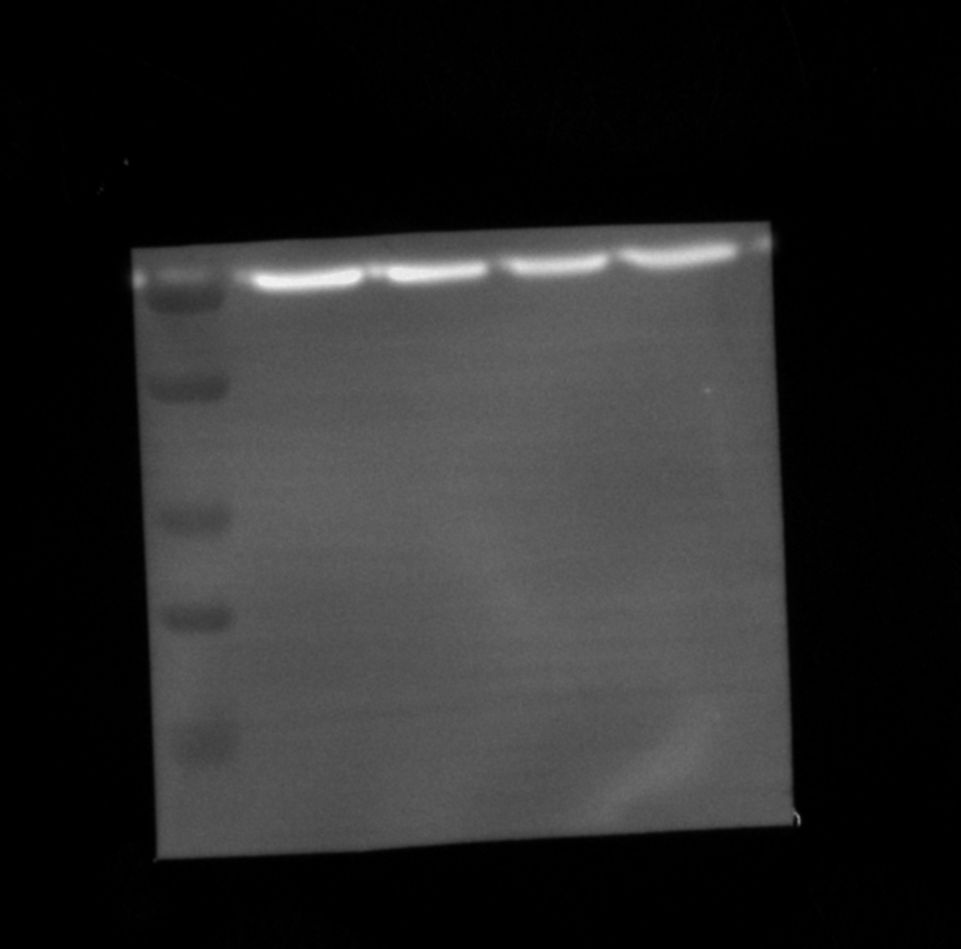

Supplement: Figure 4—source data 2. [file elife-98584-fig4-data2.zip › Figure 4 - Source Data 1/4_tub for BiP VCP w lof teri 16092021 ladder.tif]

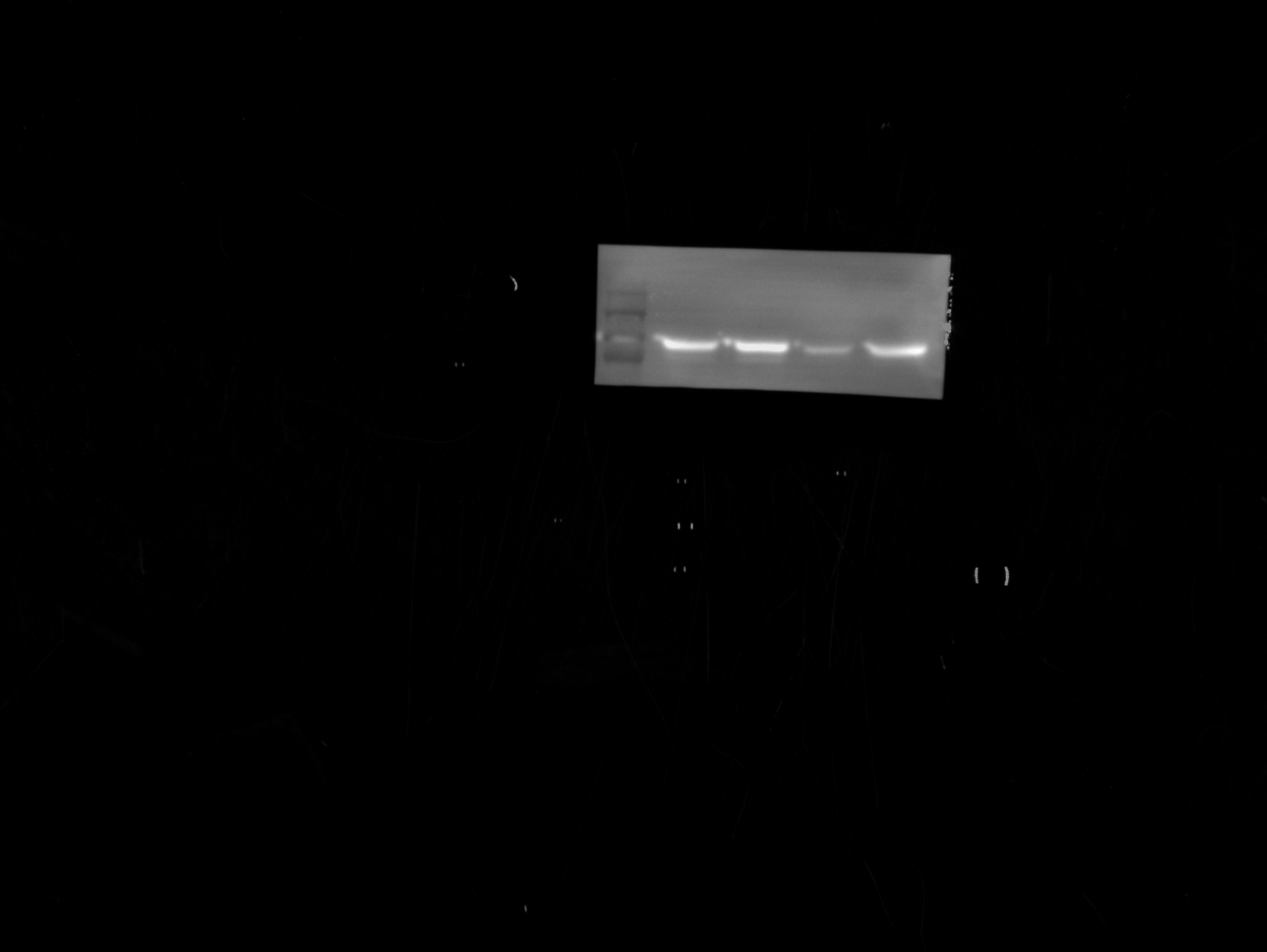

Supplement: Figure 4—source data 2. [file elife-98584-fig4-data2.zip › Figure 4 - Source Data 1/2_VCP WB w lof teri culli 16092021 ladder.tif]

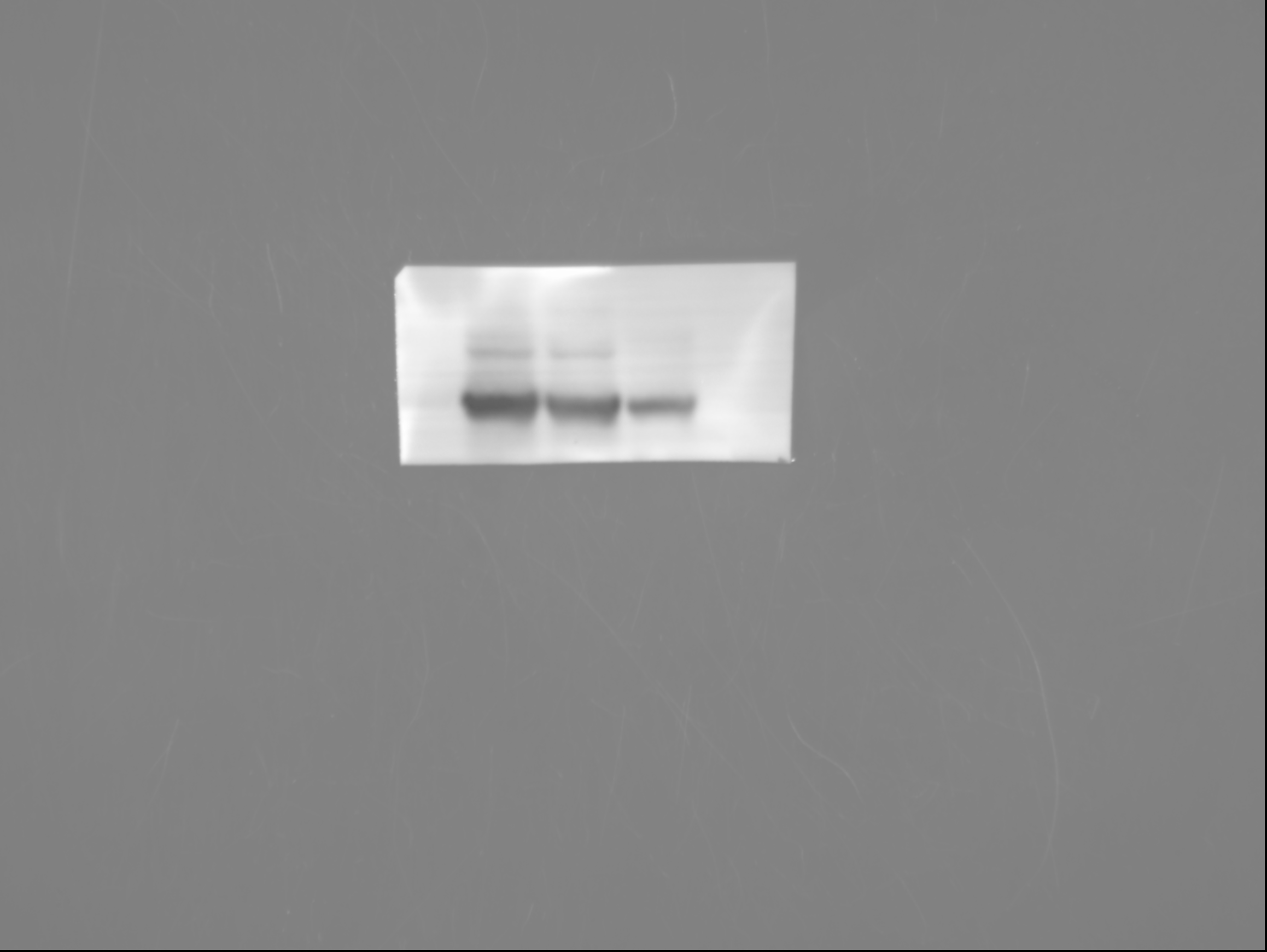

Supplement: Figure 7—source data 2. [file elife-98584-fig7-data2.zip › Figure 7- Source data 1/casp lof me31b.tif]

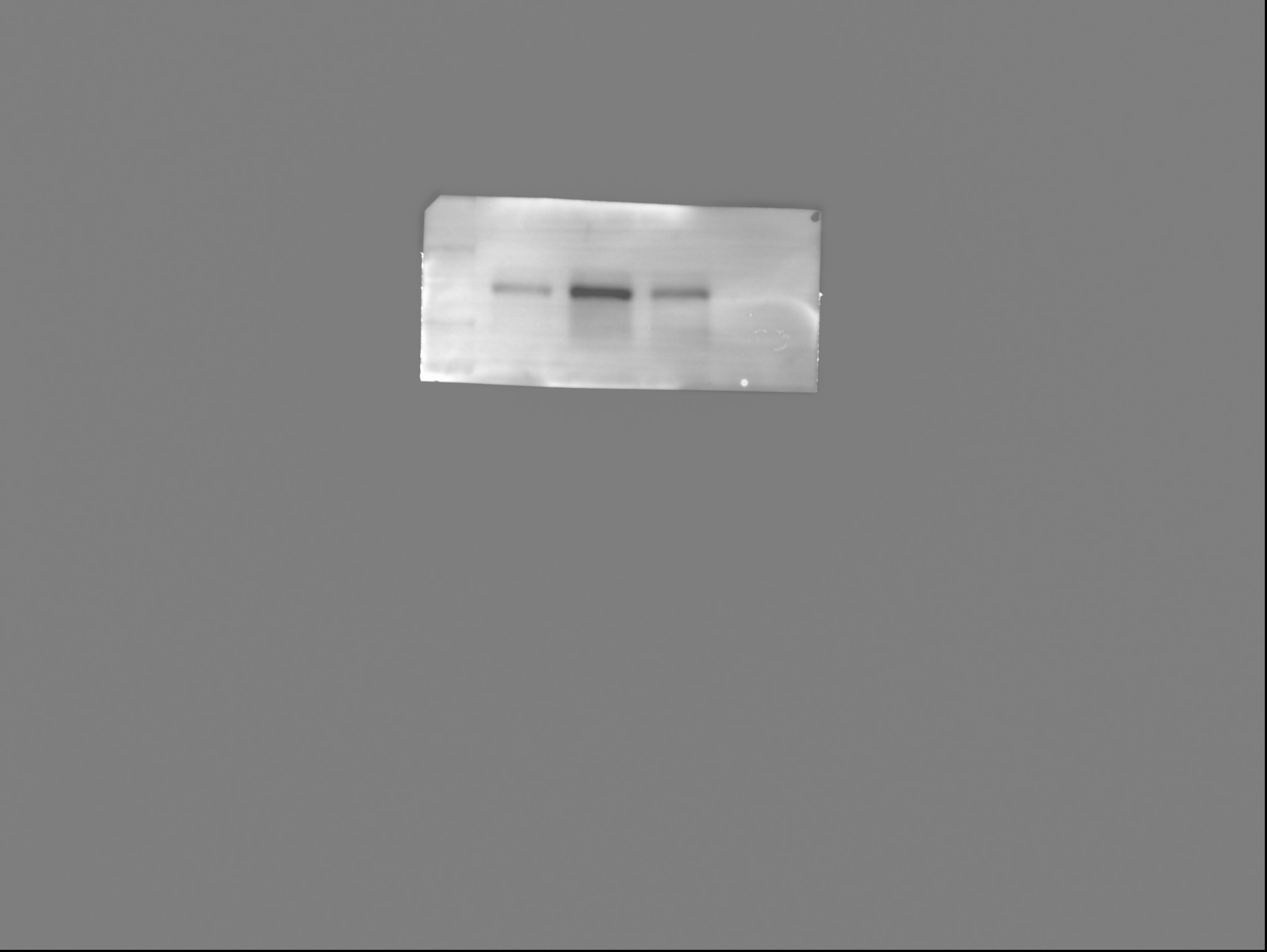

Supplement: Figure 7—source data 2. [file elife-98584-fig7-data2.zip › Figure 7- Source data 1/casp lof smaug.tif]

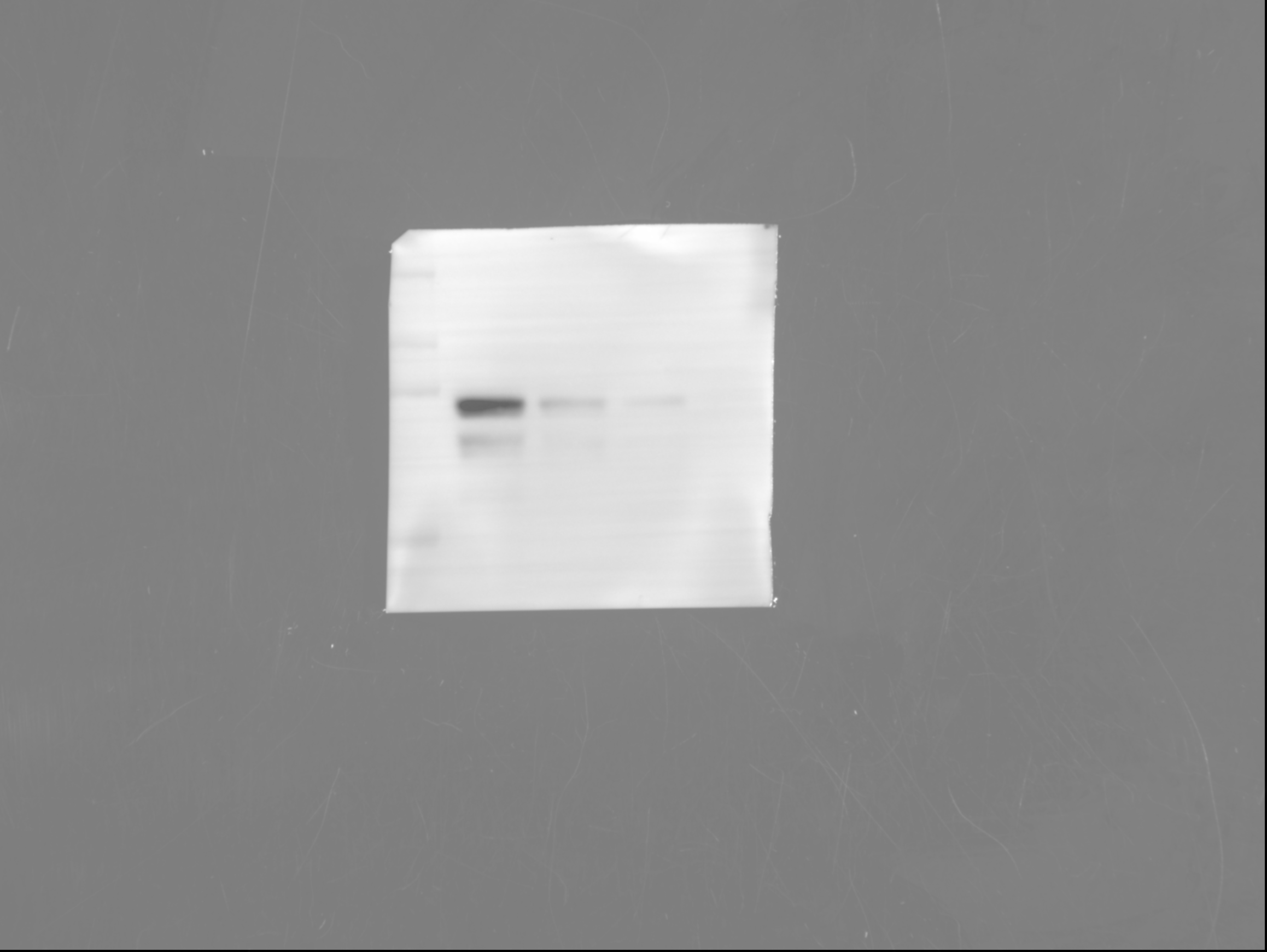

Supplement: Figure 7—source data 2. [file elife-98584-fig7-data2.zip › Figure 7- Source data 1/casp lof tral.tif]

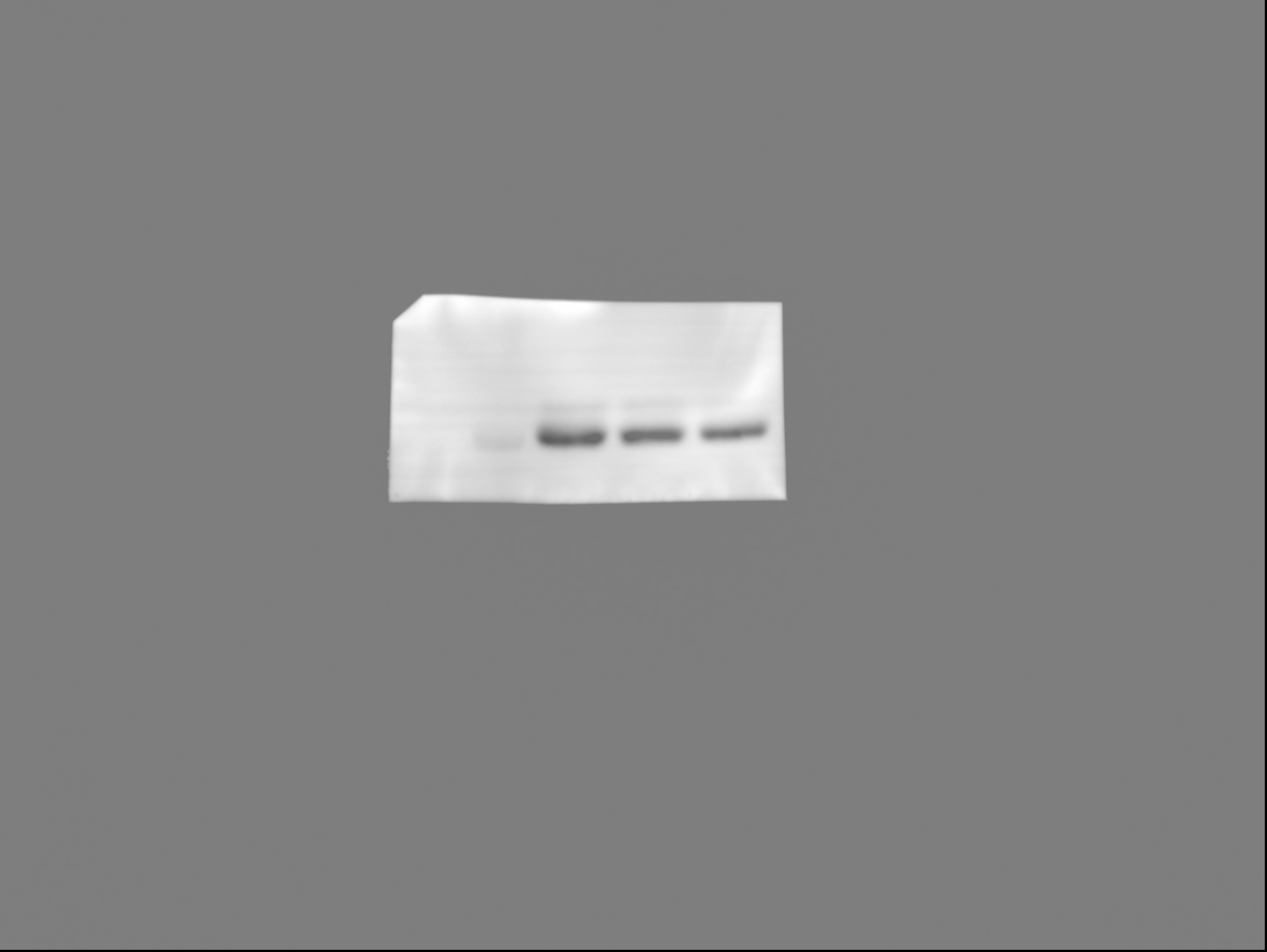

Supplement: Figure 7—source data 2. [file elife-98584-fig7-data2.zip › Figure 7- Source data 1/casp lof tubulin.tif]

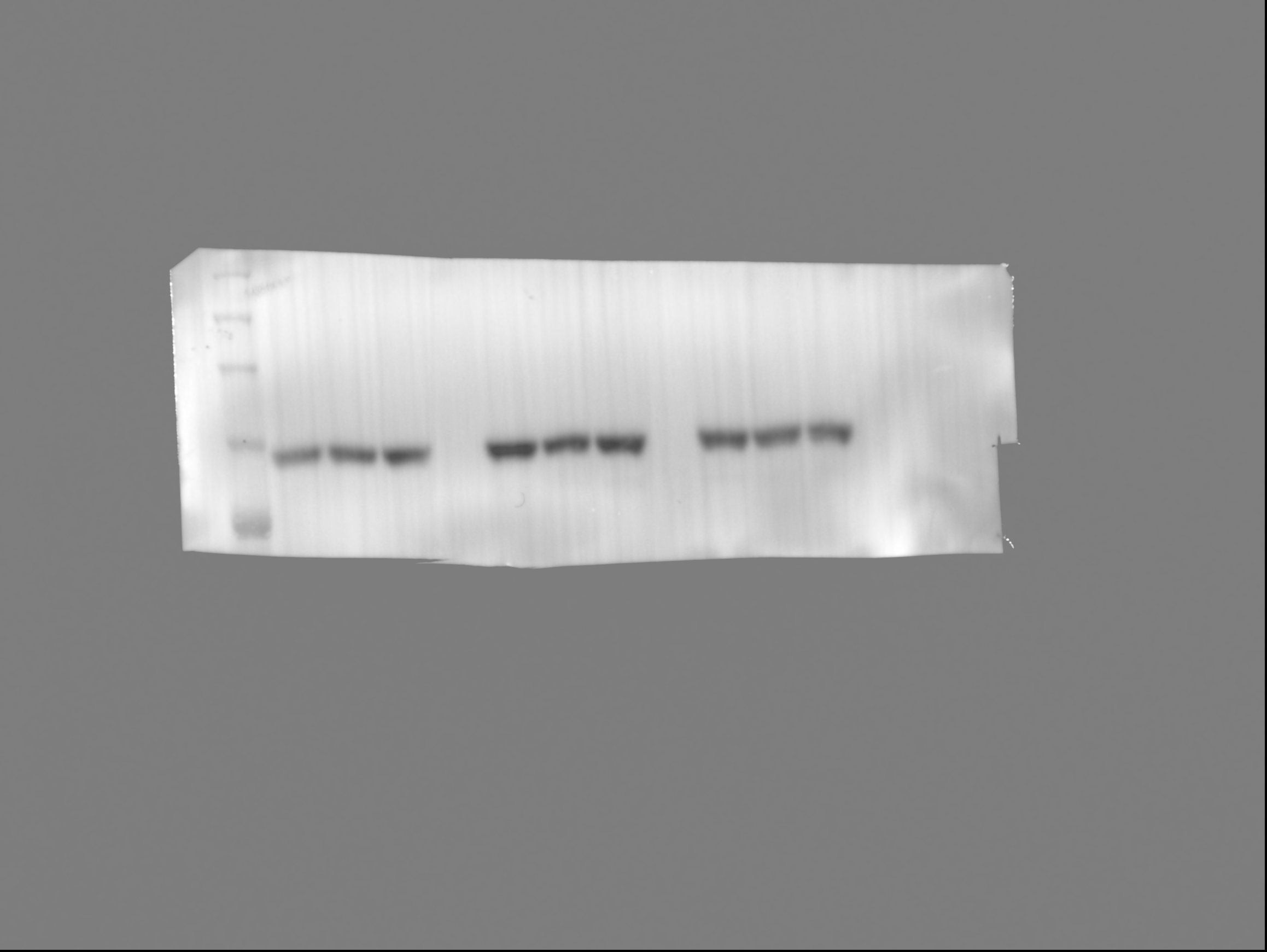

Supplement: Figure 7—source data 2. [file elife-98584-fig7-data2.zip › Figure 7- Source data 1/w1118 and casp lof ter94.tif]

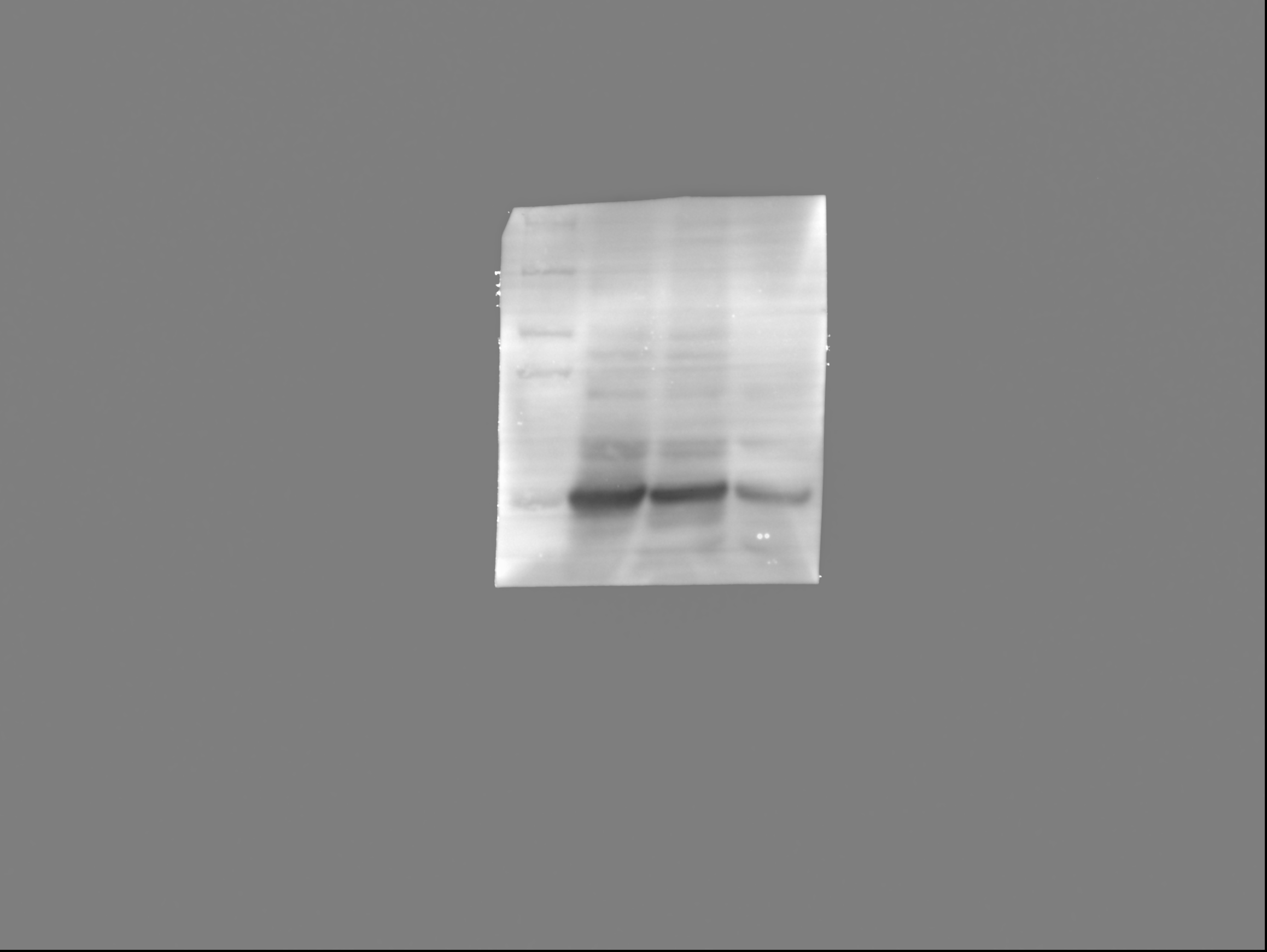

Supplement: Figure 7—source data 2. [file elife-98584-fig7-data2.zip › Figure 7- Source data 1/w1118 me31b.tif]

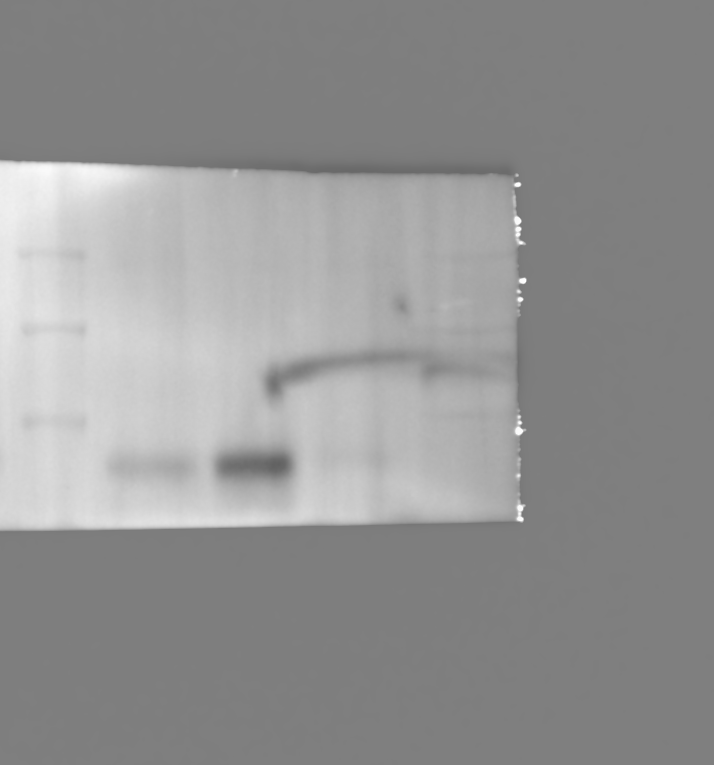

Supplement: Figure 7—source data 2. [file elife-98584-fig7-data2.zip › Figure 7- Source data 1/w1118 smaug.tif]

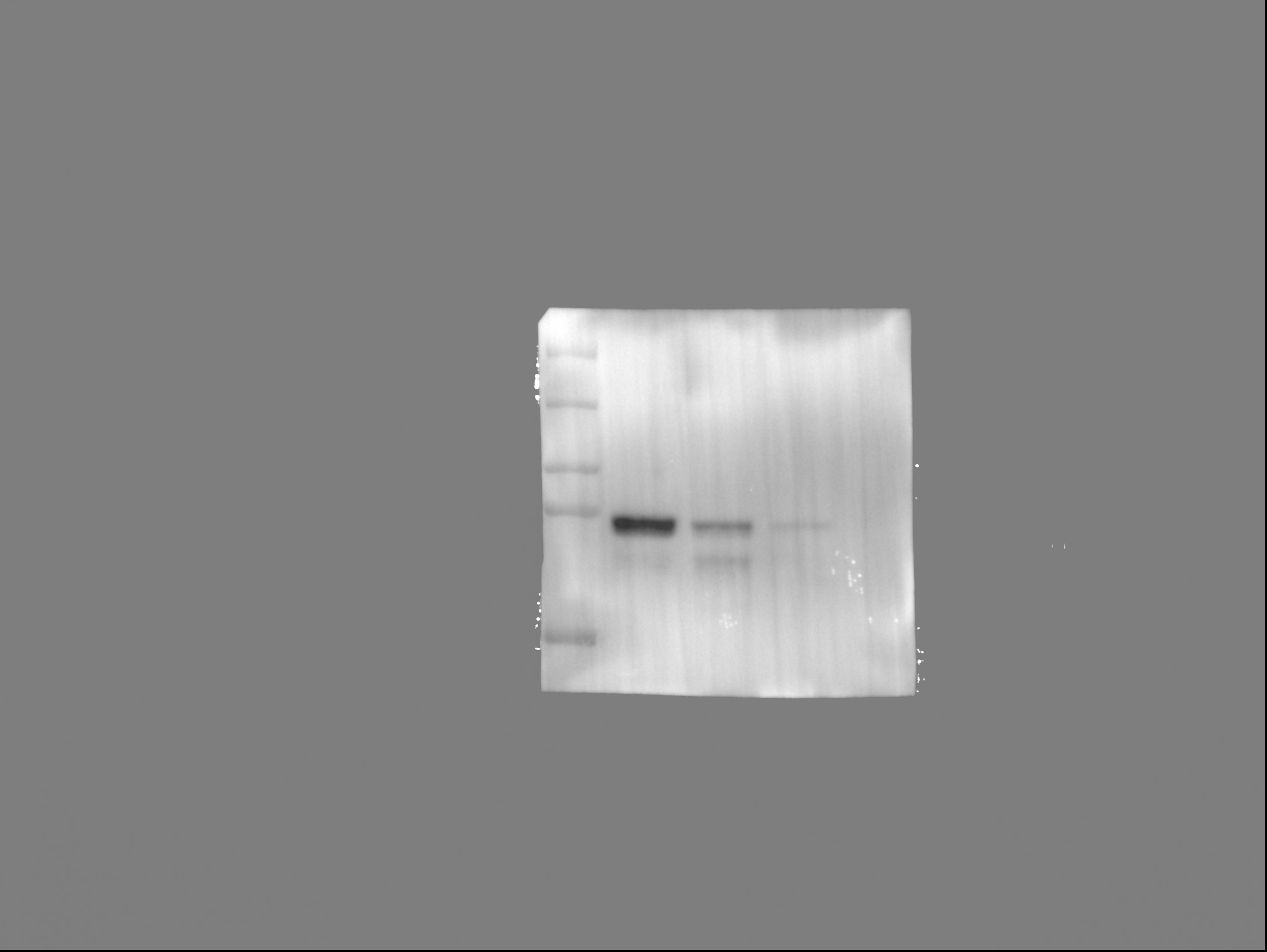

Supplement: Figure 7—source data 2. [file elife-98584-fig7-data2.zip › Figure 7- Source data 1/w1118 tral.tif]

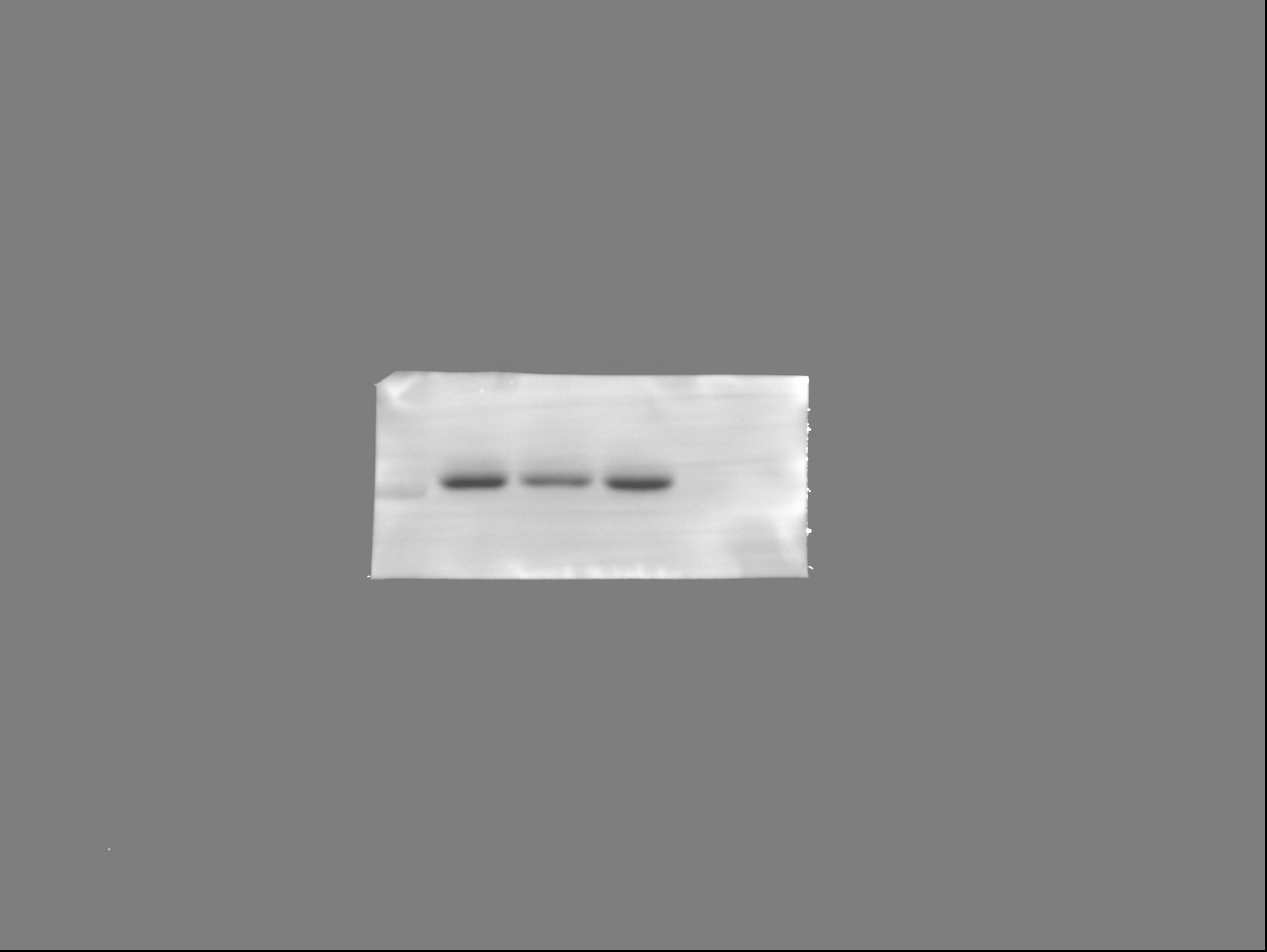

Supplement: Figure 7—source data 2. [file elife-98584-fig7-data2.zip › Figure 7- Source data 1/w1118 tubulin.tif]

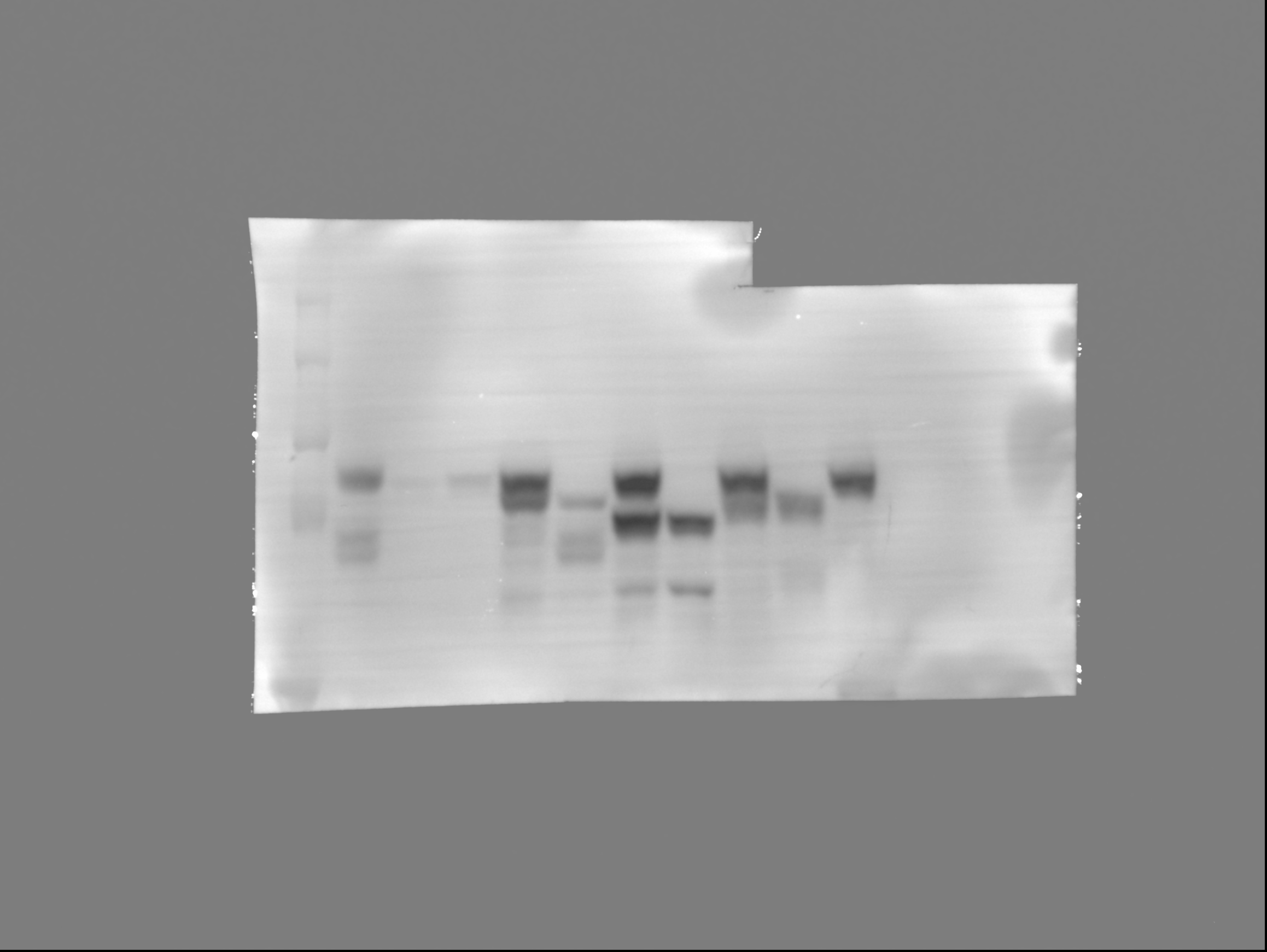

Supplement: Figure 9—source data 2. [file elife-98584-fig9-data2.zip › Figure 9- Source data 1/caspar.tif]

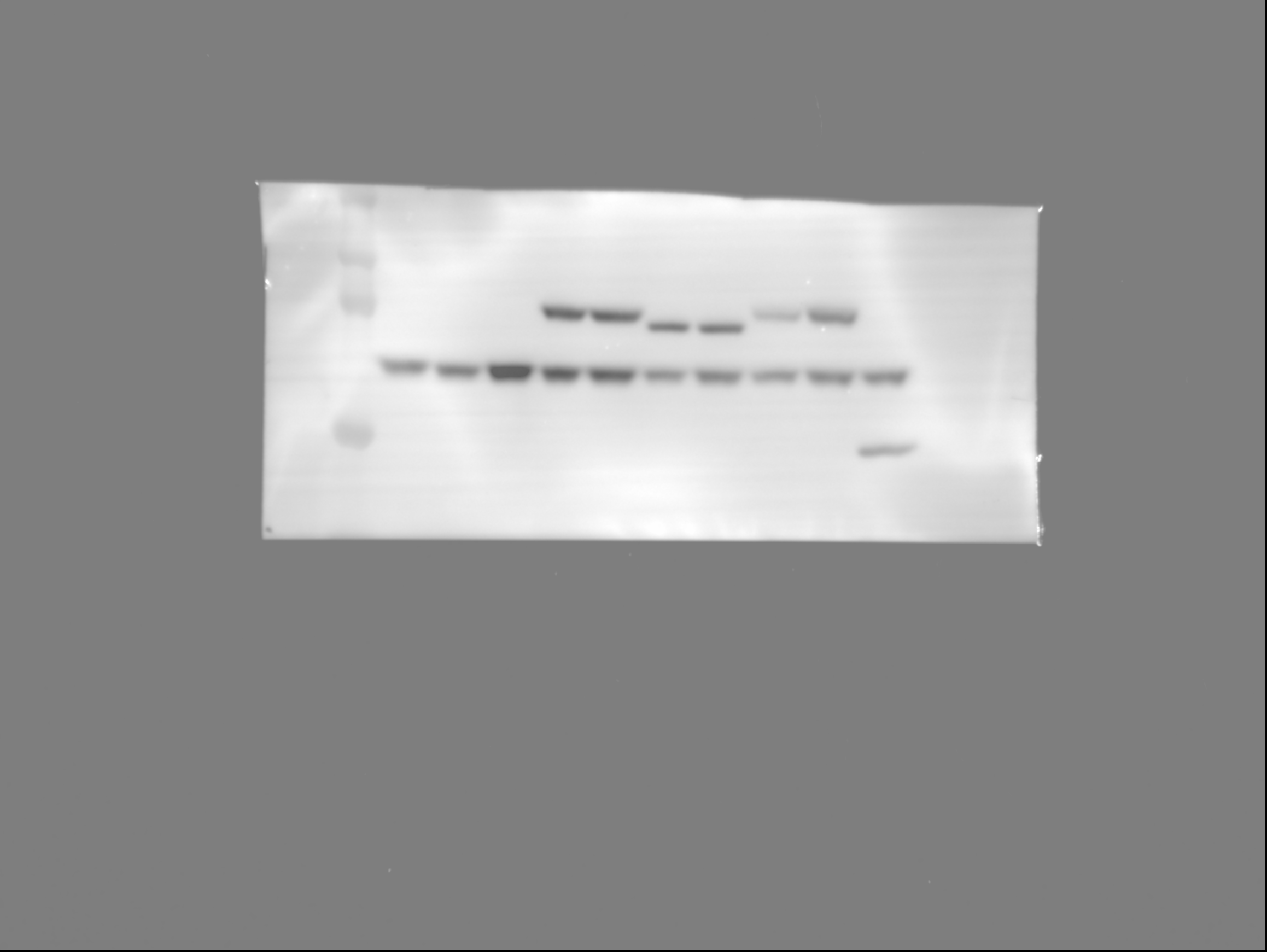

Supplement: Figure 9—source data 2. [file elife-98584-fig9-data2.zip › Figure 9- Source data 1/HA.tif]

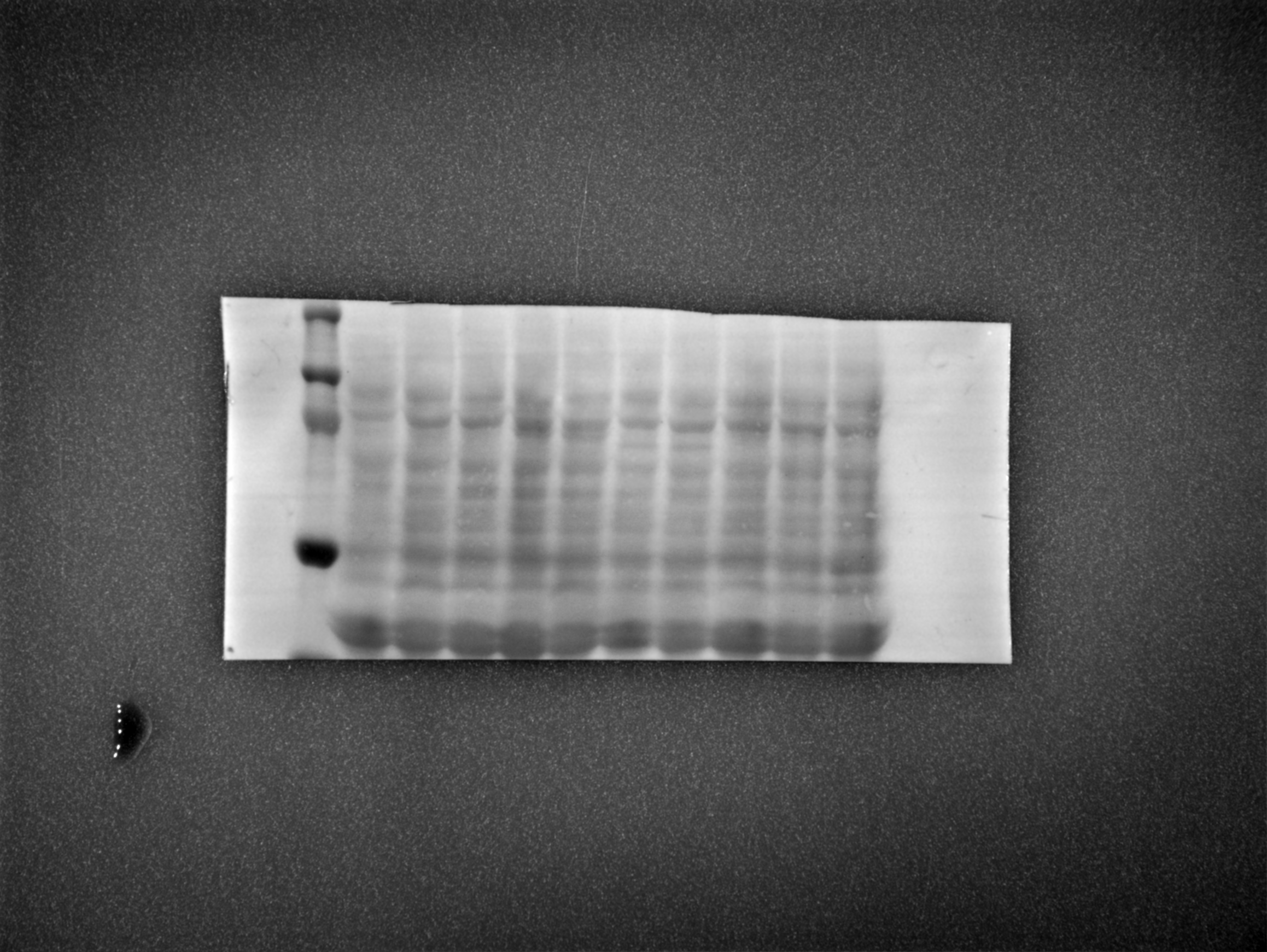

Supplement: Figure 9—source data 2. [file elife-98584-fig9-data2.zip › Figure 9- Source data 1/ponceau.tif]

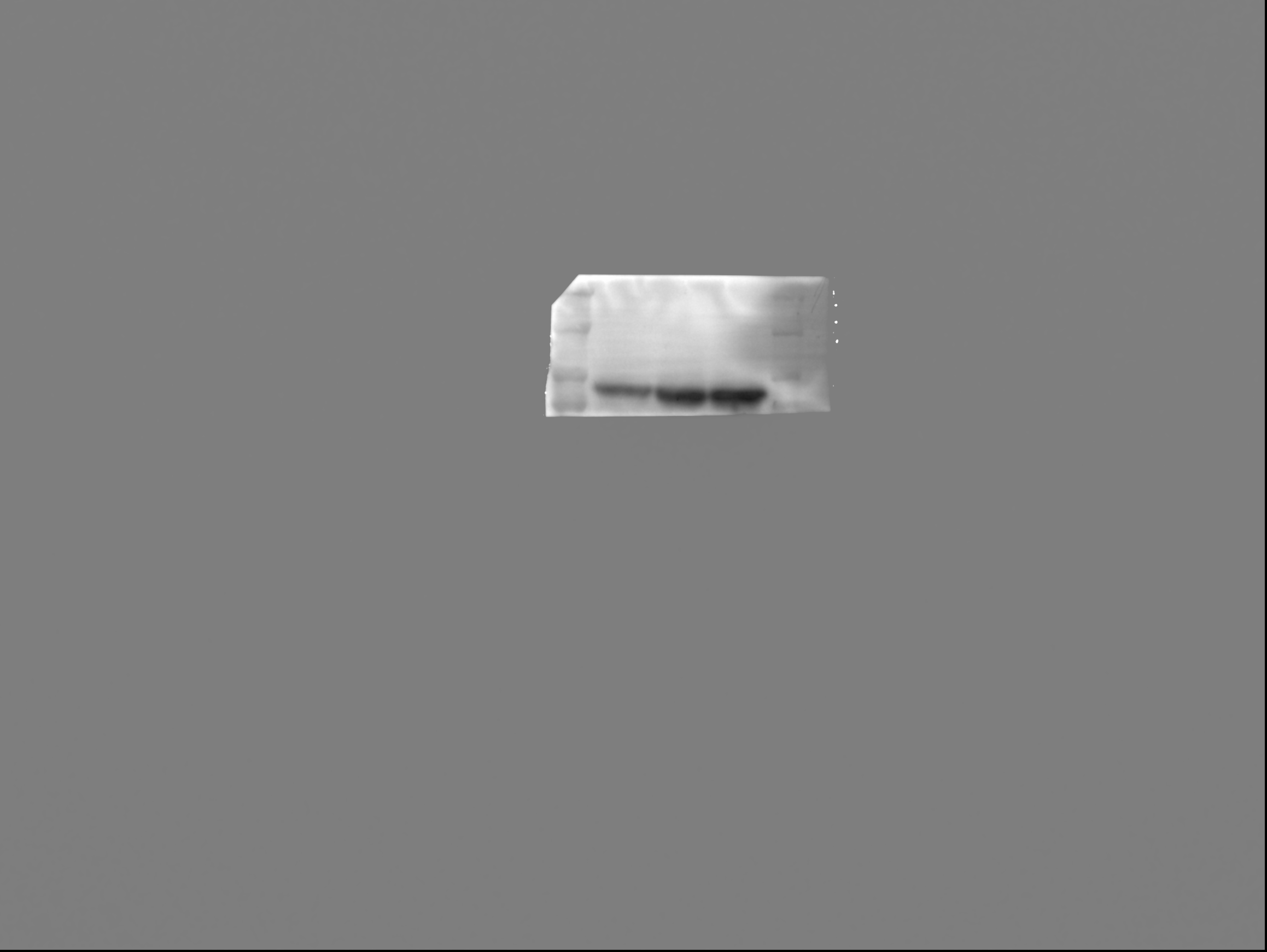

Supplement: Figure 9—figure supplement 1—source data 2. [file elife-98584-fig9-figsupp1-data2.zip › Figure 9 -Figure Supplement-source data 1/CASP FFAT.tif]

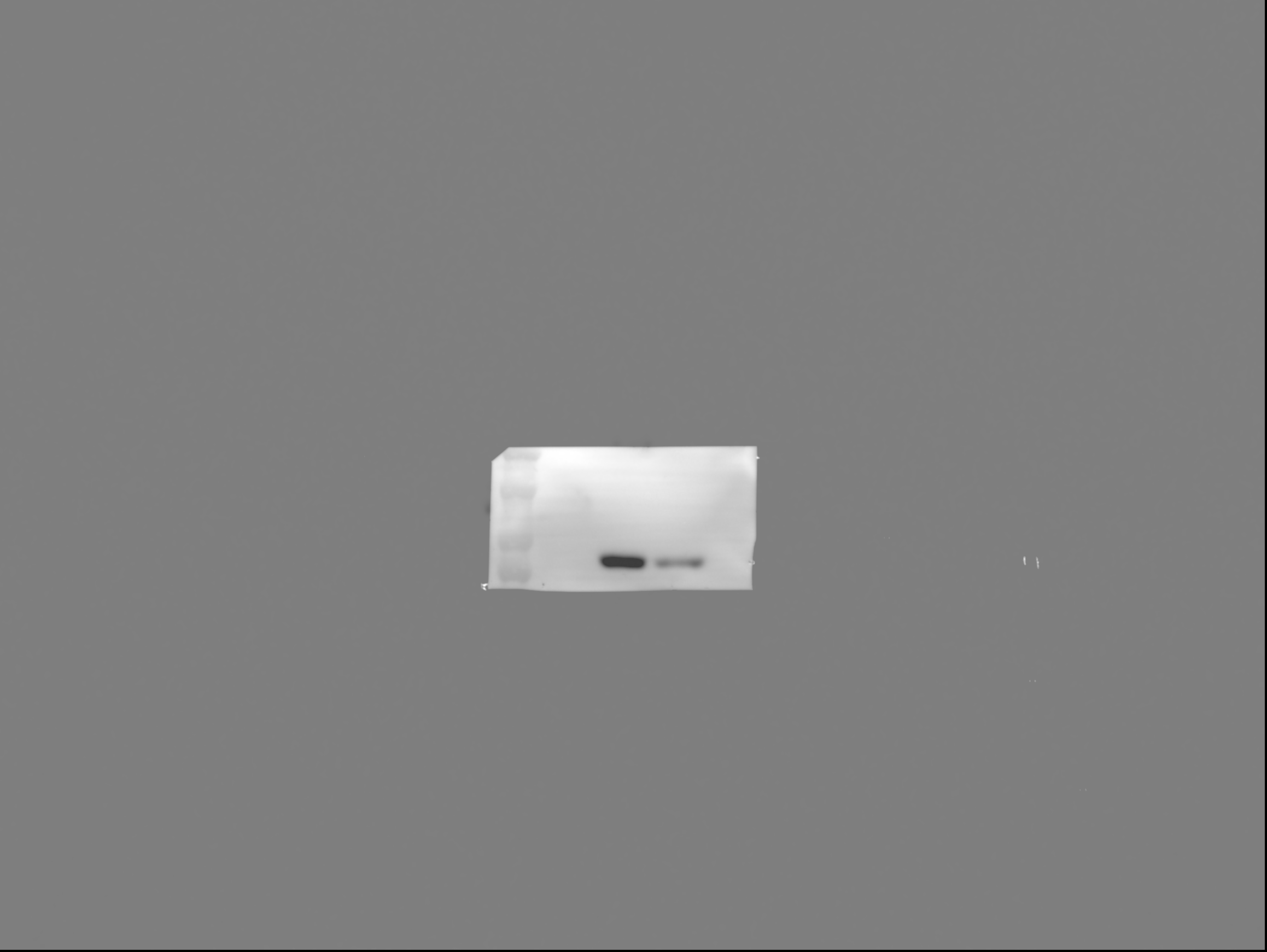

Supplement: Figure 9—figure supplement 1—source data 2. [file elife-98584-fig9-figsupp1-data2.zip › Figure 9 -Figure Supplement-source data 1/HA.tif]

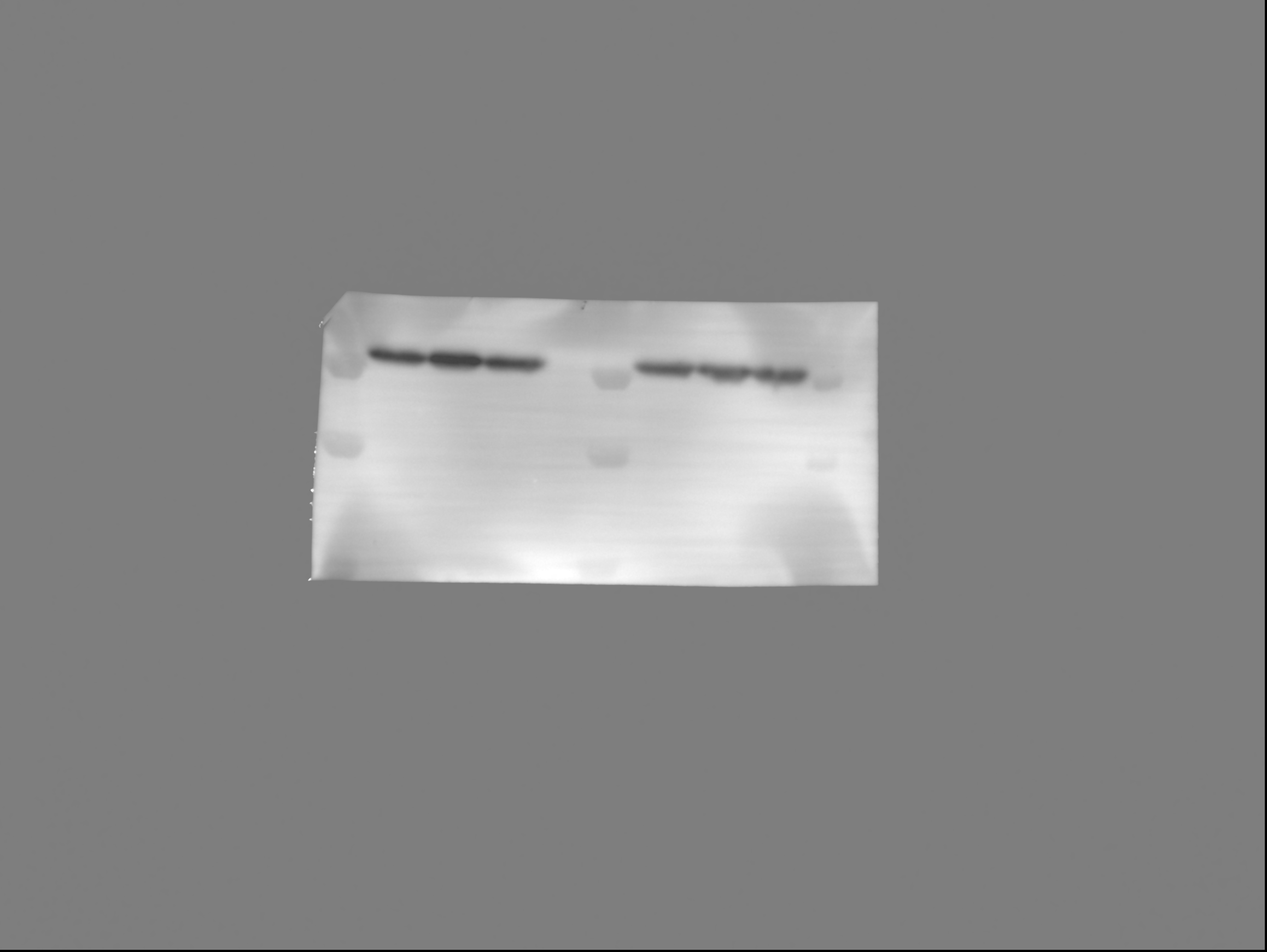

Supplement: Figure 9—figure supplement 1—source data 2. [file elife-98584-fig9-figsupp1-data2.zip › Figure 9 -Figure Supplement-source data 1/TUBULIN.tif]
