# Supplementary material for: Caspar specifies primordial germ cell count and identity in Drosophila melanogaster
Source: eLife. 2024 Dec 13;13:RP98584. doi: 10.7554/eLife.98584 (PMC11643641; doi:10.7554/eLife.98584)
Supplement: Figure 4—source data 1. [file elife-98584-fig4-data1.pdf]

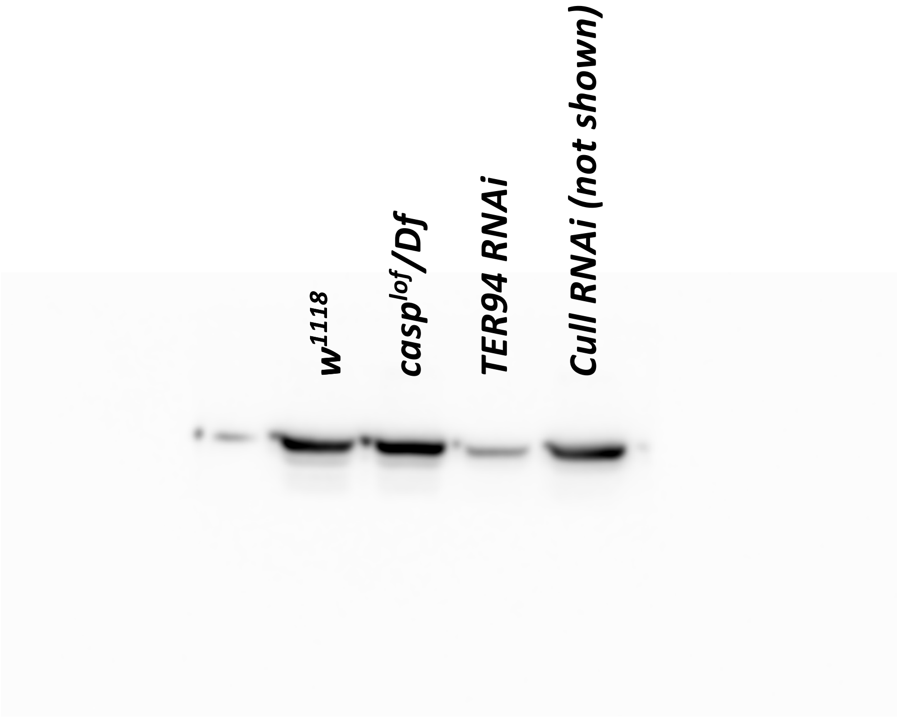


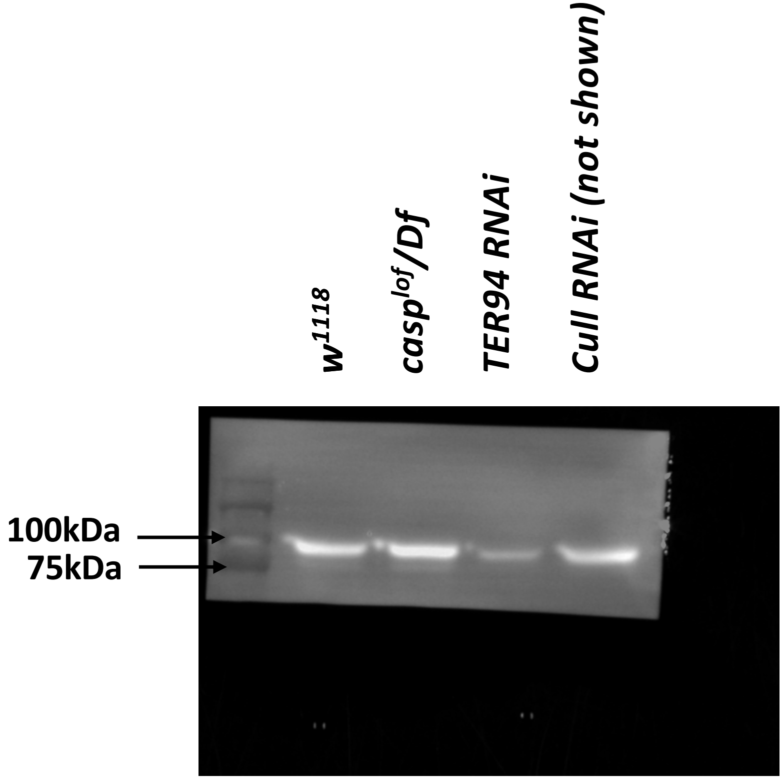


*Immunoblot: Rabbit anti Ter94 (1:2000)*

**Figure 4, Source Data 1. Original membranes corresponding to Figure 4, panel B**. Blot was probed with rabbit anti-Ter94 antibody.

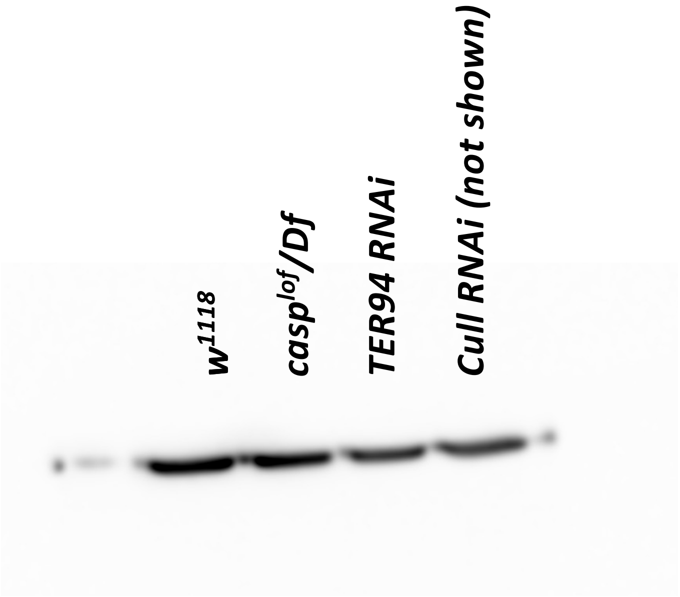


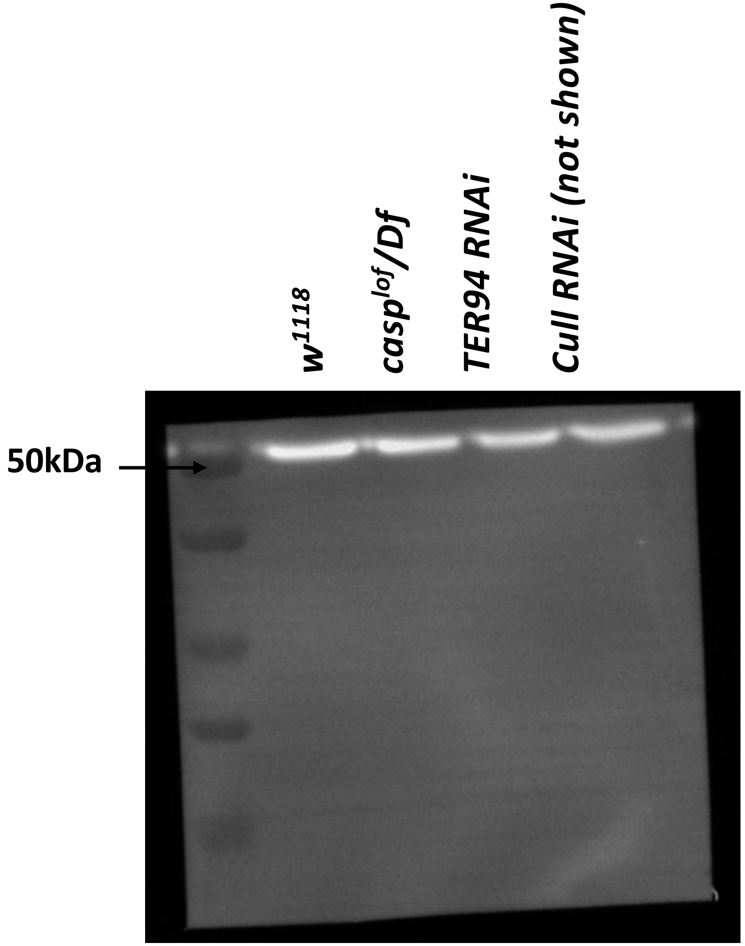


*Immunoblot: Mouse anti-Tubulin (1:10000)*

**Figure 4, Source Data 1. Original membranes corresponding to Figure 4, panel B**. Blot was probed with Mouse anti-Tubulin antibody.
