# Supplementary material for: Caspar specifies primordial germ cell count and identity in Drosophila melanogaster
Source: eLife. 2024 Dec 13;13:RP98584. doi: 10.7554/eLife.98584 (PMC11643641; doi:10.7554/eLife.98584)
Supplement: Figure 7—source data 1. [file elife-98584-fig7-data1.pdf]

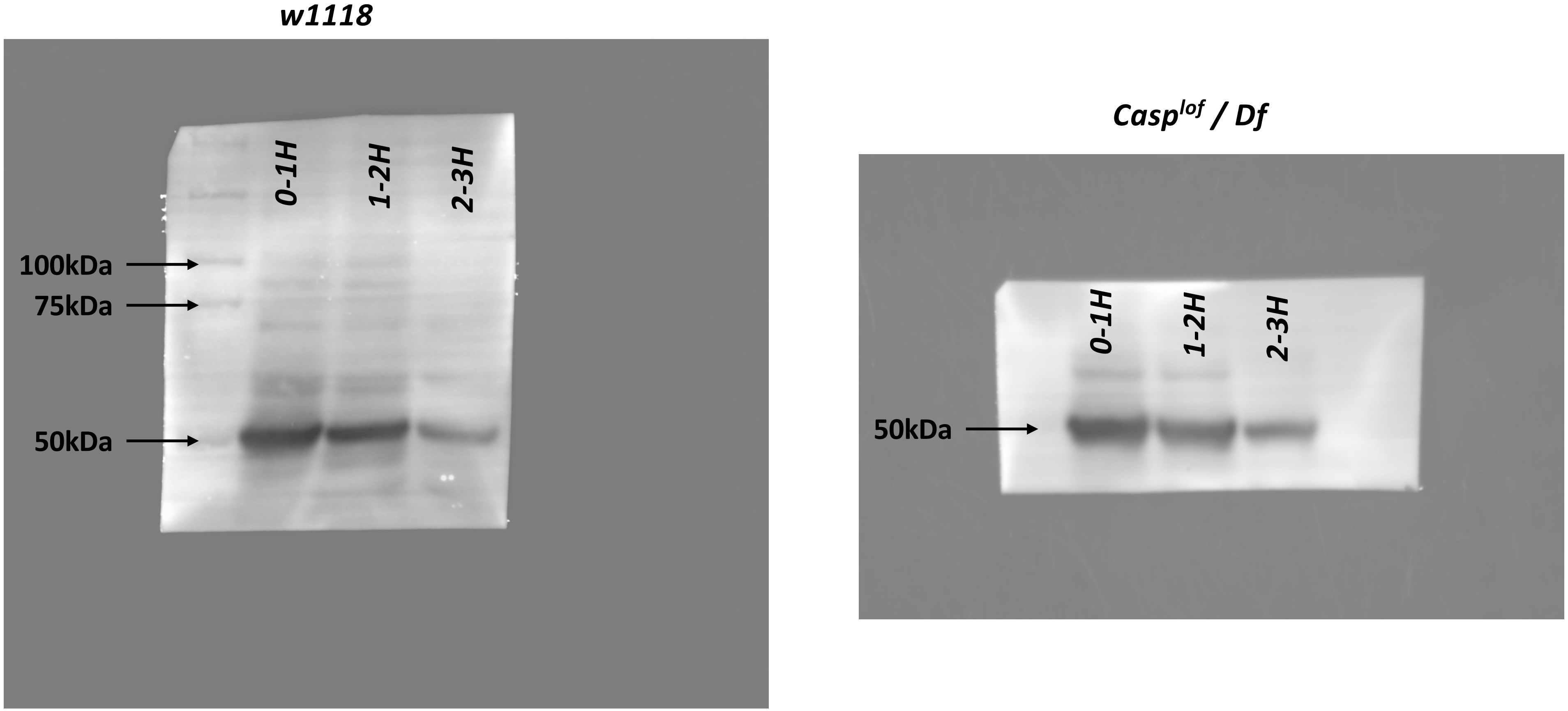

Immunoblot: Rabbit anti Me31B (1:1000)

Figure 7, Source Data 1. Original membranes corresponding to Figure 7, panel A1 and A2. Blot was probed with rabbit anti-Me31B antibody.

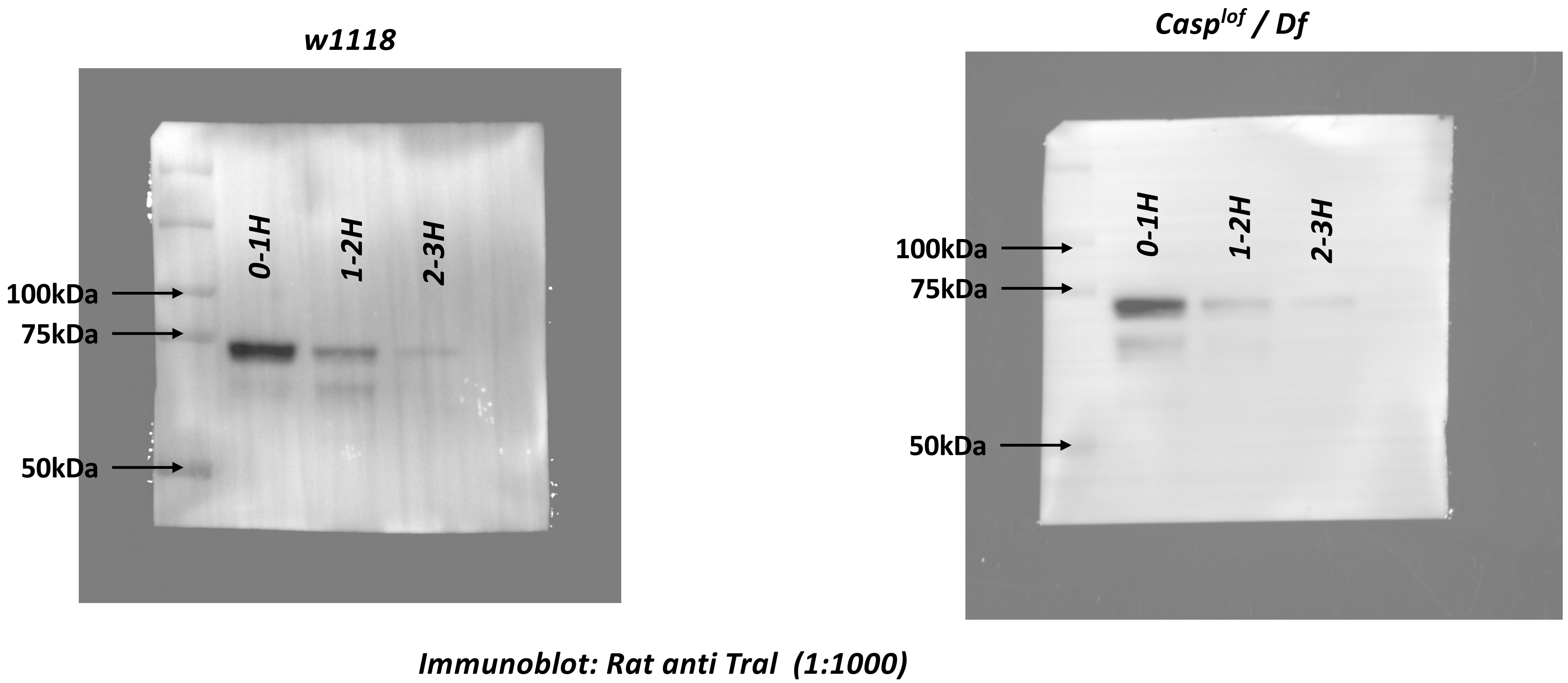

Figure 7, Source Data 1. Original membranes corresponding to Figure 7, panel B1 and B2. Blot was probed with rat anti-Tral antibody.

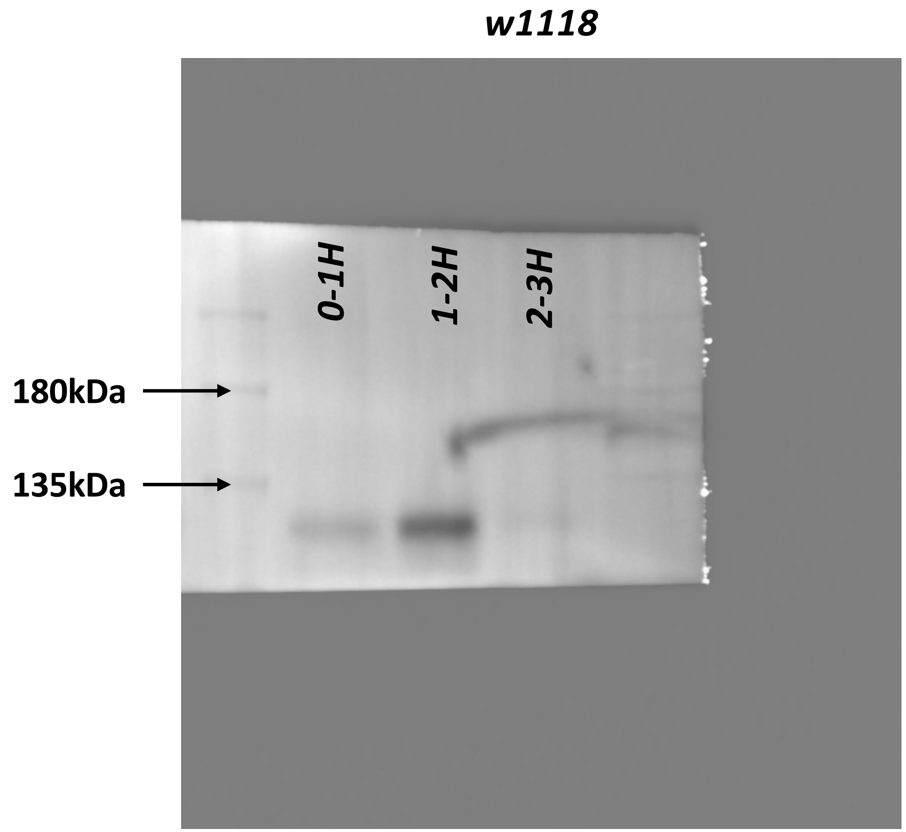

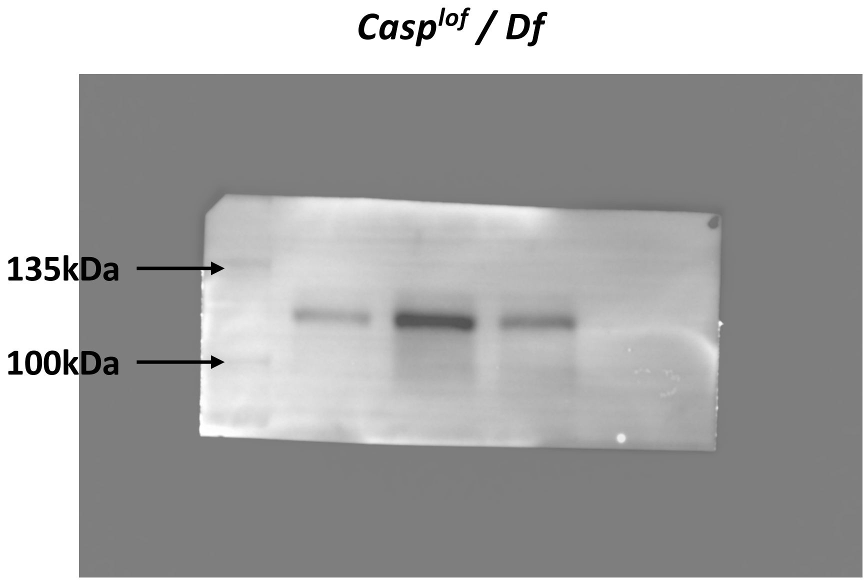

Immunoblot: Rabbit anti smaug (1:200)

Figure 7, Source Data 1. Original membranes corresponding to Figure 7, panel C1 and C2. Blot was probed with rabbit anti-Smaug antibody.

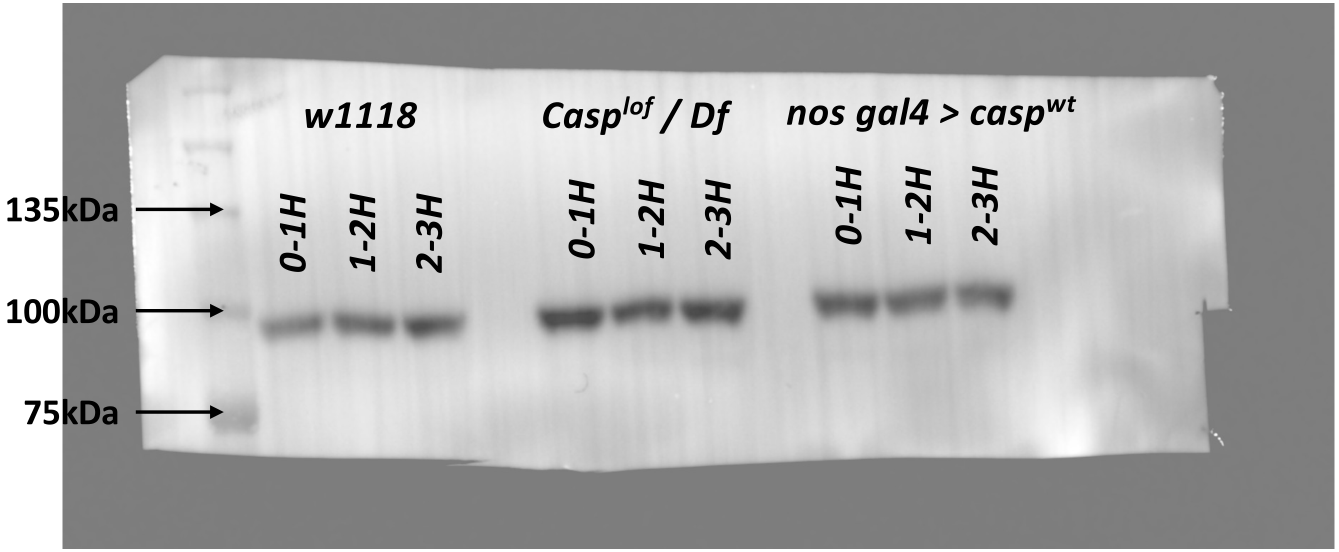

Immunoblot: Rabbit anti Ter94 (1:2000)

Figure 7, Source Data 1. Original membranes corresponding to Figure 7, panel D1 and D2. Blot was probed with rabbit anti-Ter94 antibody. Ter94 levels in ***nos gal4 > casp^{wt}*** is not shown the paper.

w1118

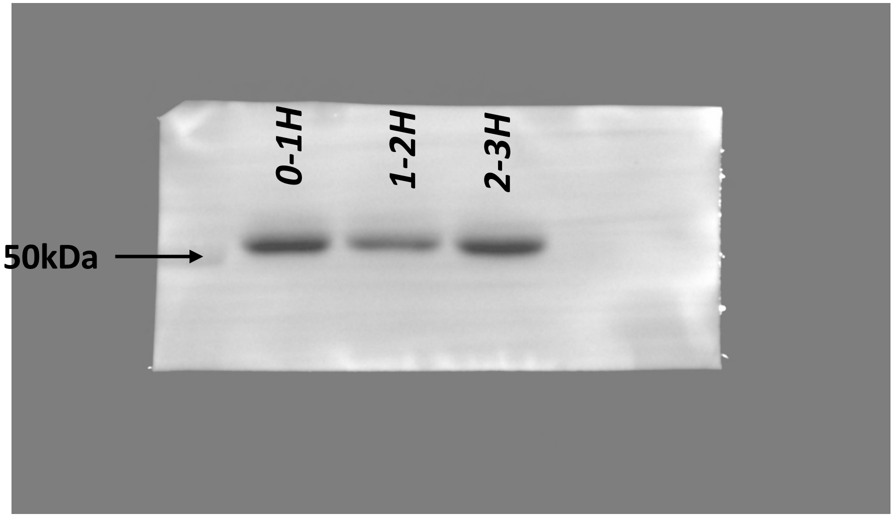

$Casp^{lof}$ / Df

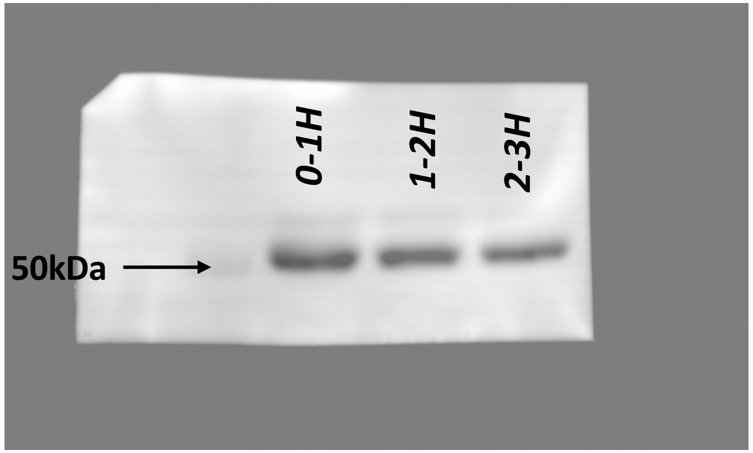

Immunoblot: Mouse anti Tubulin (1:10,000)

Figure 7, Source Data 1. Original membranes corresponding to Figure 7, panel E1 and E2. Blot was probed with mouse anti-Tubulin antibody.
