# Supplementary material for: Caspar specifies primordial germ cell count and identity in Drosophila melanogaster
Source: eLife. 2024 Dec 13;13:RP98584. doi: 10.7554/eLife.98584 (PMC11643641; doi:10.7554/eLife.98584)
Supplement: Figure 9—source data 1. [file elife-98584-fig9-data1.pdf]

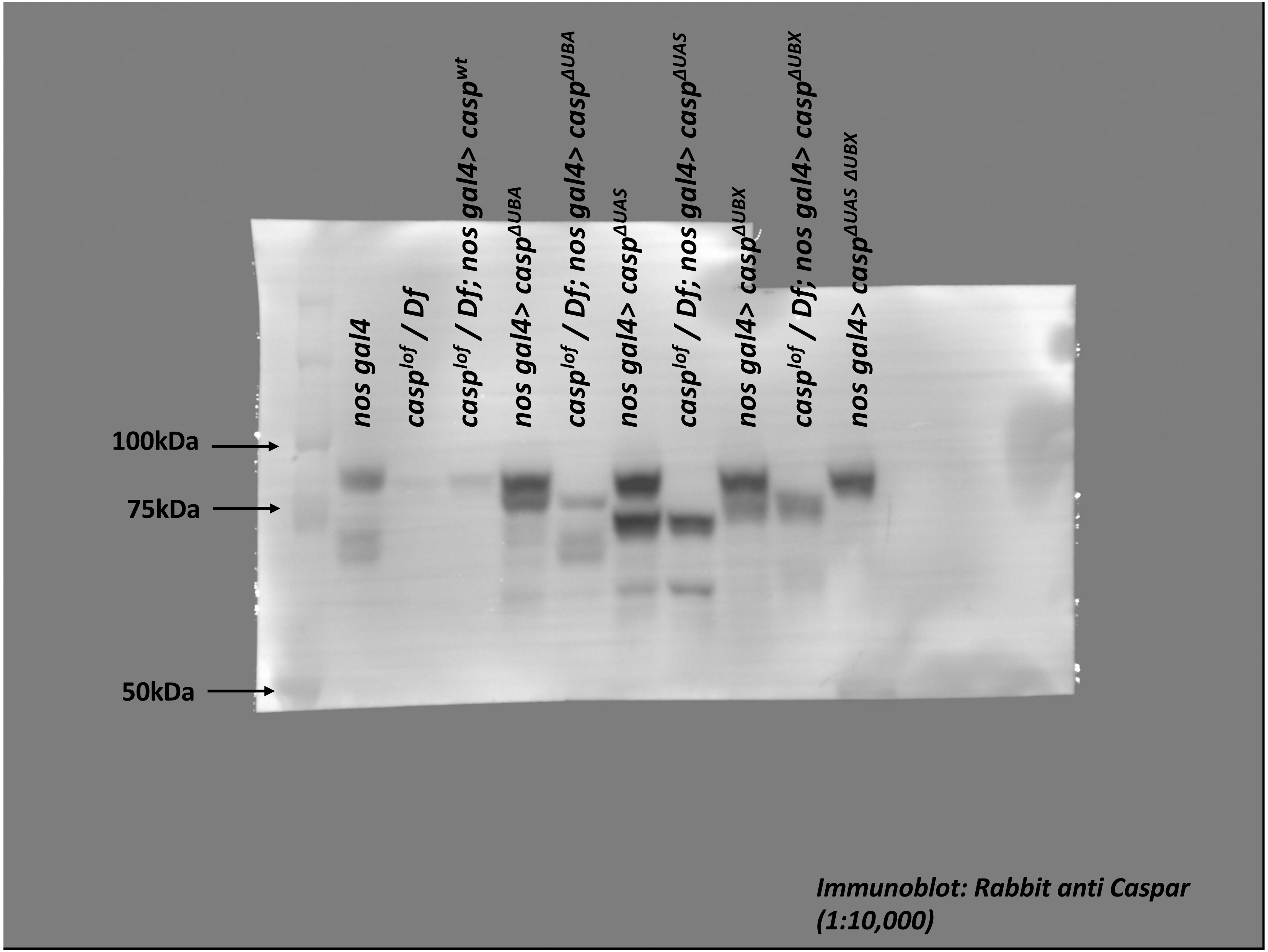

Figure 9, Source Data 1. Original membranes corresponding to Figure 9, panel B. Blot was probed with rabbit anti-Caspar antibody. Biorad Precision Plus Protein standards (Dual Colour) was used as molecular weight marker

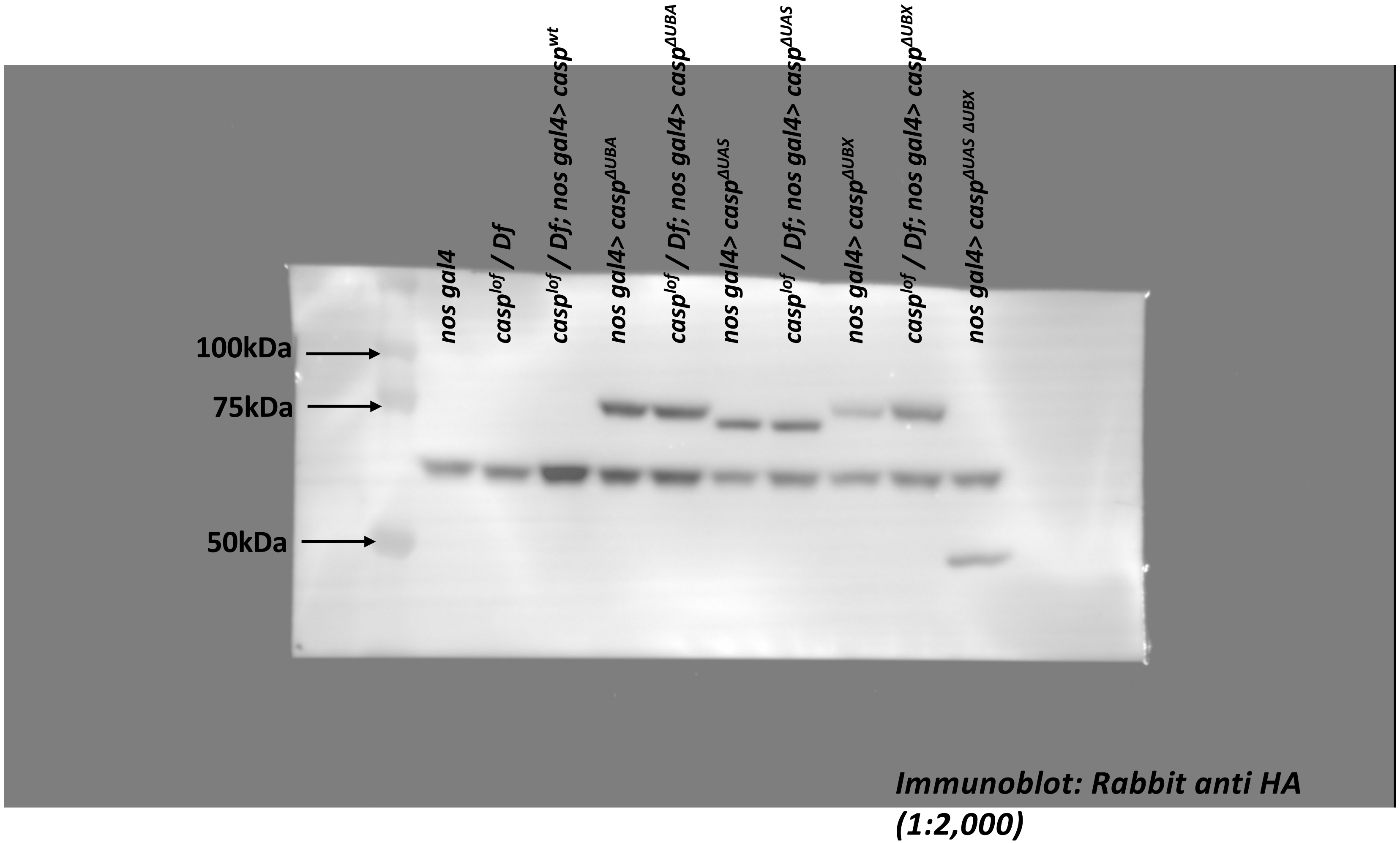

Figure 9, Source Data 1. Original membranes corresponding to Figure 9, panel B. Blot was probed with rabbit anti-HA antibody. Biorad Precision Plus Protein standards (Dual Colour) was used as molecular weight marker

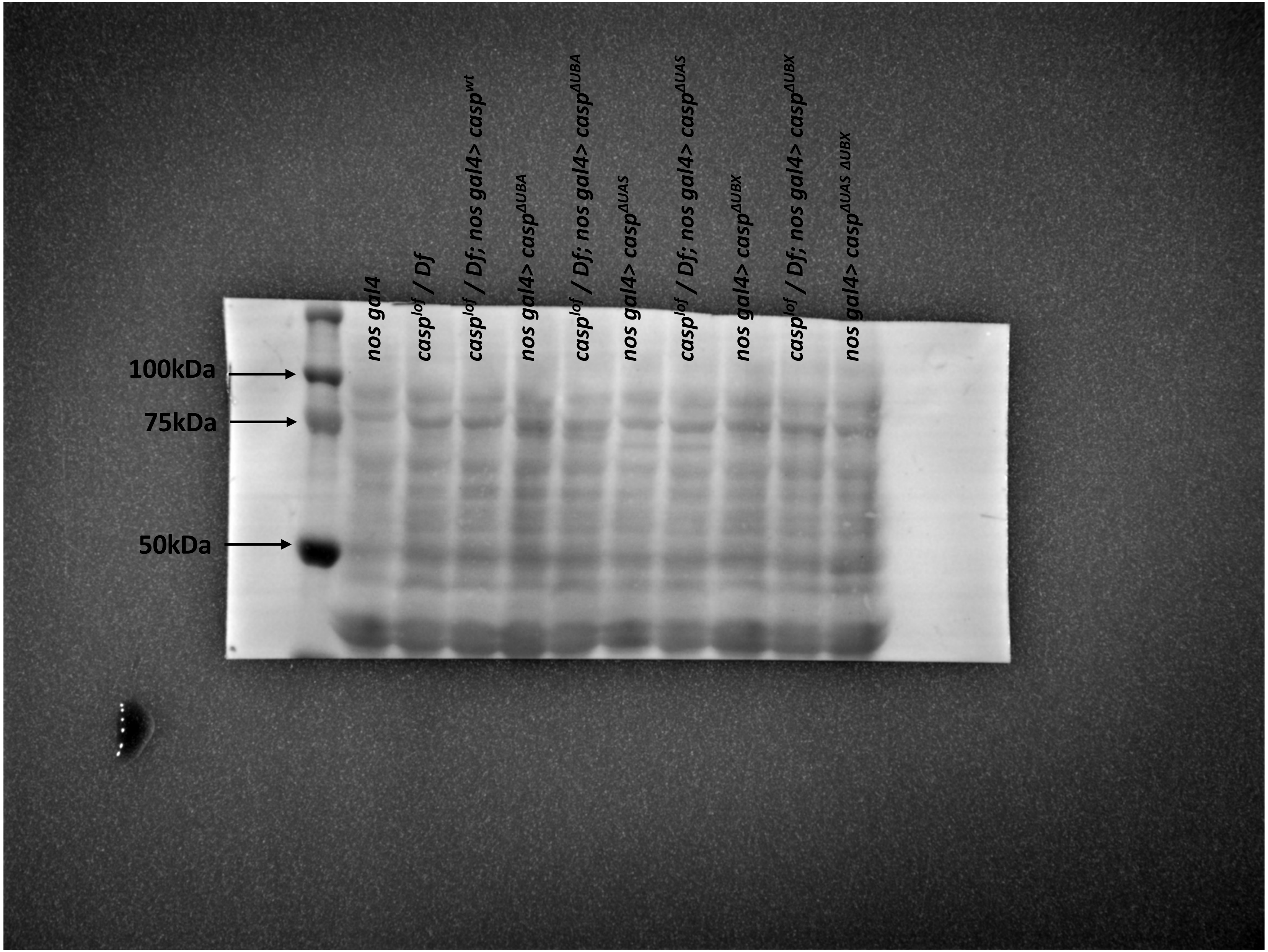

Figure 9, Source Data 1. Original membranes corresponding to Figure 9, panel B. Blot represents Ponceau staining to show equal protein loading. Biorad Precision Plus Protein standards (Dual Colour) was used as molecular weight marker

Ponceau-s
