# Supplementary material for: Caspar specifies primordial germ cell count and identity in Drosophila melanogaster
Source: eLife. 2024 Dec 13;13:RP98584. doi: 10.7554/eLife.98584 (PMC11643641; doi:10.7554/eLife.98584)
Supplement: Figure 9—figure supplement 1—source data 1. [file elife-98584-fig9-figsupp1-data1.pdf]

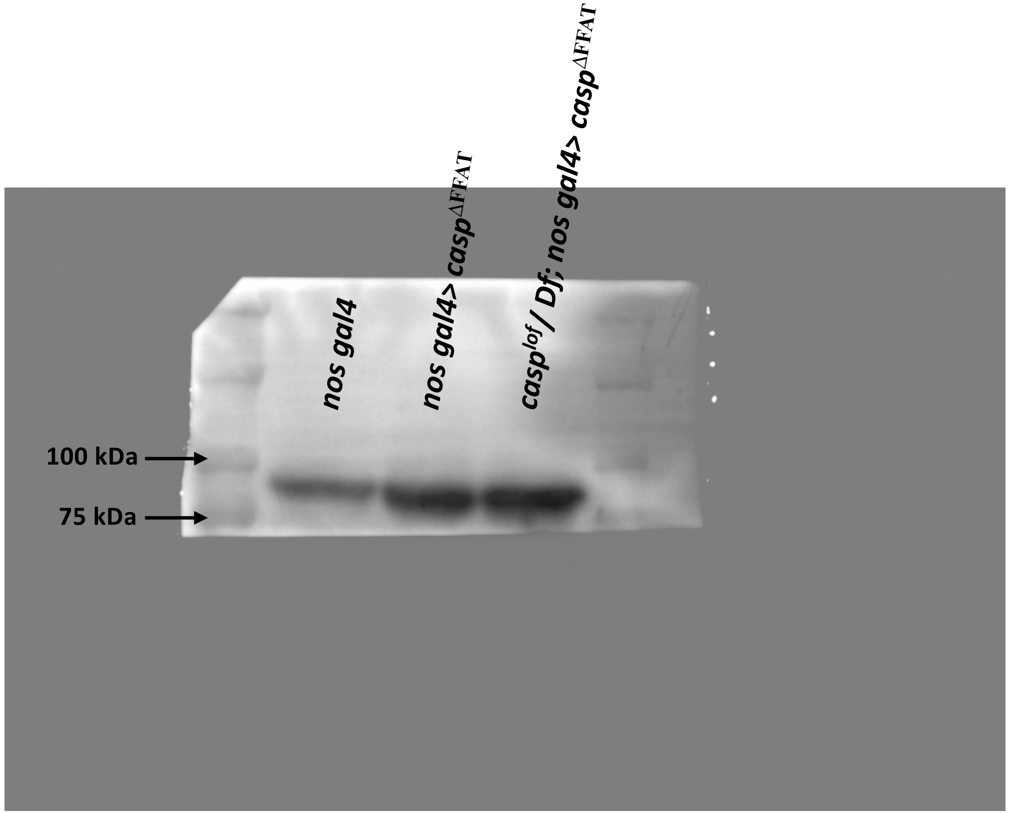

Immunoblot: Rabbit anti-Caspar (1:10,000)

Figure 9, Supplementary Source Data 1. Original membranes corresponding to Figure 9, Supplementary 1 panel C. Blot was probed with rabbit anti-Caspar antibody. Biorad Precision Plus Protein standards (Dual Colour) was used as molecular weight marker

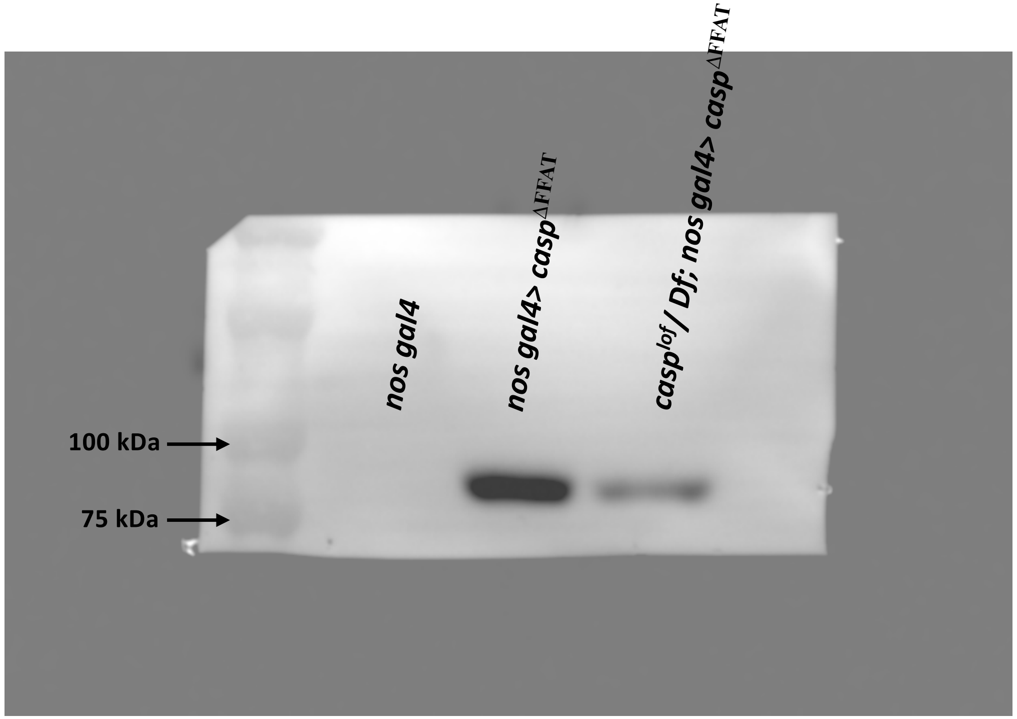

Immunoblot: Rabbit anti-HA (1:2000)

Figure 9, Supplementary Source Data 1. Original membranes corresponding to Figure 9, Supplementary 1 panel C. Blot was probed with rabbit anti-HA antibody. Biorad Precision Plus Protein standards (Dual Colour) was used as molecular weight marker

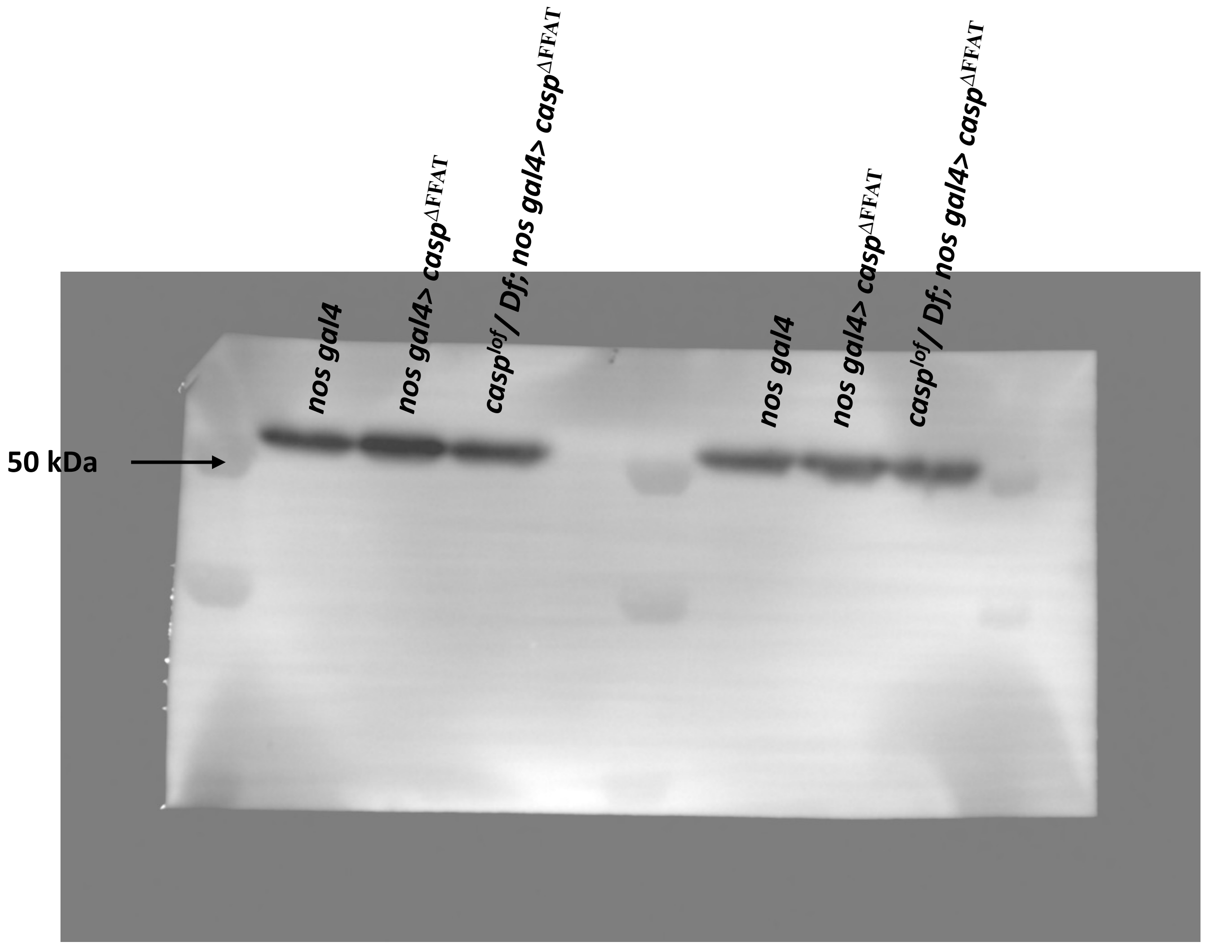

Immunoblot: Mouse anti-Tubulin (1:10,000)

Figure 9, Supplementary Source Data 1. Original membranes corresponding to Figure 9, Supplementary 1 panel C. Blot was probed with mouse anti-tubulin antibody. Biorad Precision Plus Protein standards (Dual Colour) was used as molecular weight marker
