# Supplementary material for: Caspar specifies primordial germ cell count and identity in Drosophila melanogaster
Source: eLife. 2024 Dec 13;13:RP98584. doi: 10.7554/eLife.98584 (PMC11643641; doi:10.7554/eLife.98584)
Supplement: MDAR checklist [file elife-98584-mdarchecklist1.pdf]

eLife

# <u>M</u>aterials <u>D</u>esign <u>A</u>nalysis <u>R</u>eporting (MDAR) Checklist for Authors

The MDAR framework establishes a minimum set of requirements in transparent reporting mainly applicable to studies in the life sciences.

*eLife* asks authors to **provide detailed information within their article** to facilitate the interpretation and replication of their work. Authors can also upload supporting materials to comply with relevant reporting guidelines for health-related research (see EQUATOR Network), life science research (see the BioSharing Information Resource), or animal research (see the ARRIVE Guidelines and the STRANGE Framework; for details, see *eLife*'s Journal Policies). Where applicable, authors should refer to any relevant reporting standards materials in this form.

For all that apply, please note **where in the article** the information is provided. Please note that we also collect information about data availability and ethics in the submission form.

## Materials:

| Newly created materials | Indicate where provided: section/figure legend | N/A |
|---|---|---|
| The manuscript includes a dedicated "materials availability statement" providing transparent disclosure about availability of newly created materials including details on how materials can be accessed and describing any restrictions on access. | The ‘Materials and methods’ section describes all materials used with part numbers. There are no restrictions on any materials created in our laboratory. | |

| Antibodies | Indicate where provided: section/figure legend | N/A |
|---|---|---|
| For commercial reagents, provide supplier name, catalogue number and RRID, if available. | The ‘Materials and methods’ section lists all commercial regents with part number and supplier name. Reagents available in-house will be shared freely with Academic Laboratories. | |

| DNA and RNA sequences | Indicate where provided: section/figure legend | N/A |
|---|---|---|
| Short novel DNA or RNA including primers, probes: Sequences should be included or deposited in a public repository. | Primers used for cloning and PCR are described in ‘Materials and methods’ section. Sequences are included within the section. | |

| Cell materials | Indicate where provided: section/figure legend | N/A |
|---|---|---|

| | | |
|---|---|---|
| Cell lines: Provide species information, strain. Provide accession number in repository OR supplier name, catalog number, clone number, OR RRID. | Not used in study | N/A |
| Primary cultures: Provide species, strain, sex of origin, genetic modification status. | Not used in study | N/A |

| **Experimental animals** | **Indicate where provided: section/figure legend** | **N/A** |
|---|---|---|
| Laboratory animals or Model organisms: Provide species, strain, sex, age, genetic modification status. Provide accession number in repository OR supplier name, catalog number, clone number, OR RRID. | Drosophila melanogaster strains used are defined in ‘Materials and methods’ section, along with supplier name and line number. | |
| Animal observed in or captured from the field: Provide species, sex, and age where possible. | Not applicable | N/A |

| **Plants and microbes** | **Indicate where provided: section/figure legend** | **N/A** |
|---|---|---|
| Plants: provide species and strain, ecotype and cultivar where relevant, unique accession number if available, and source (including location for collected wild specimens). | Not applicable | N/A |
| Microbes: provide species and strain, unique accession number if available, and source. | Not applicable | N/A |

| **Human research participants** | **Indicate where provided: section/figure legend) or state if these demographics were not collected** | **N/A** |
|---|---|---|
| If collected and within the bounds of privacy constraints report on age, sex, gender and ethnicity for all study participants. | Not applicable | **N/A** |

## Design:

| **Study protocol** | **Indicate where provided: section/figure legend** | **N/A** |
|---|---|---|
| If the study protocol has been pre-registered, provide DOI. For clinical trials, provide the trial registration number OR cite DOI. | Not applicable | N/A |

| **Laboratory protocol** | **Indicate where provided: section/figure legend** | **N/A** |
|---|---|---|
| Provide DOI OR other citation details if detailed step-by-step protocols are available. | Not applicable | |

| **Experimental study design (statistics details) *** | | |
|---|---|---|
| **For in vivo studies: State whether and how the following have been done** | **Indicate where provided: section/figure legend. If it could have been done, but was not, write “not done”** | **N/A** |
| Sample size determination | Materials and methods and figure legends. For western blots N (Number of replicates)=3. For pole cell count N~30 embryos. | |
| Randomisation | Not applicable | **N/A** |
| Blinding | Not applicable | N/A |
| Inclusion/exclusion criteria | Not applicable | N/A |

| **Sample definition and in-laboratory replication** | **Indicate where provided: section/figure legend** | **N/A** |
|---|---|---|
| State number of times the experiment was replicated in the laboratory. | Figure legends describe number of biological (N) and technical (n) replicates. | |
| Define whether data describe technical or biological replicates. | Figure legends (N defines biological replicates, n defines technical replicates) | |

| **Ethics** | **Indicate where provided: section/submission form** | **N/A** |
|---|---|---|
| Studies involving human participants: State details of authority granting ethics approval (IRB or equivalent committee(s), provide reference number for approval. | Not applicable | N/A |
| Studies involving experimental animals: State details of authority granting ethics approval (IRB or equivalent committee(s), provide reference number for approval. | IISER Institutional Biosafety Committee (IBSC) | |

| | | |
|---|---|---|
| Studies involving specimen and field samples: State if relevant permits obtained, provide details of authority approving study; if none were required, explain why. | Not applicable | N/A |

| **Dual Use Research of Concern (DURC)** | **Indicate where provided: section/submission form** | **N/A** |
|---|---|---|
| If study is subject to dual use research of concern regulations, state the authority granting approval and reference number for the regulatory approval. | Not applicable | N/A |

## Analysis:

| **Attrition** | **Indicate where provided: section/figure legend** | **N/A** |
|---|---|---|
| Describe whether exclusion criteria were pre-established. Report if sample or data points were omitted from analysis. If yes, report if this was due to attrition or intentional exclusion and provide justification. | Not applicable | N/A |

| **Statistics** | **Indicate where provided: section/figure legend** | **N/A** |
|---|---|---|
| Describe statistical tests used and justify choice of tests. | Statistical tests are described in Figure legends. | |

| **Data availability** | **Indicate where provided: section/submission form** | **N/A** |
|---|---|---|
| For newly created and reused datasets, the manuscript includes a data availability statement that provides details for access (or notes restrictions on access). | All data is within the Manuscript and Supplementary material. Large datasets were not generated. | N/A |
| When newly created datasets are publicly available, provide accession number in repository OR DOI and licensing details where available. | Not applicable | N/A |
| If reused data is publicly available provide accession number in repository OR DOI, OR URL, OR citation. | Not applicable | |

| **Code availability** | **Indicate where provided: section/figure legend** | **N/A** |
|---|---|---|

| | | |
|---|---|---|
| For any computer code/software/mathematical algorithms essential for replicating the main findings of the study, whether newly generated or re-used, the manuscript includes a data availability statement that provides details for access or notes restrictions. | Not applicable | N/A |
| Where newly generated code is publicly available, provide accession number in repository, OR DOI OR URL and licensing details where available. State any restrictions on code availability or accessibility. | Not applicable | N/A |
| If reused code is publicly available provide accession number in repository OR DOI OR URL, OR citation. | Not applicable | N/A |

## Reporting:

The MDAR framework recommends adoption of discipline-specific guidelines, established and endorsed through community initiatives.

| **Adherence to community standards** | **Indicate where provided: section/figure legend** | **N/A** |
|---|---|---|
| State if relevant guidelines (e.g., ICMJE, MIBBI, ARRIVE, STRANGE) have been followed, and whether a checklist (e.g., CONSORT, PRISMA, ARRIVE) is provided with the manuscript. | Flybase nomenclature is used for *Drosophila* strains or gene names. | |
